# The scalable growth of high-performance nanostructured heterojunction photoanodes for applications in tandem photoelectrochemical-photovoltaic solar water splitting devices†

**DOI:** 10.1039/d4sc08595g

**Published:** 2025-04-01

**Authors:** Brian Tam, Sebastian D. Pike, Jenny Nelson, Andreas Kafizas

**Affiliations:** a Department of Chemistry, Molecular Science Research Hub, Imperial College London White City London W12 0BZ UK b.tam18@imperial.ac.uk; b Department of Physics, Imperial College London South Kensington London SW7 2AZ UK; c Department of Chemistry, University of Warwick Coventry CV4 7AL UK; d London Centre for Nanotechnology, Imperial College London South Kensington London SW7 2AZ UK

## Abstract

Due to their complementary absorption characteristics and band energy structure, the BiVO_4_-coated WO_3_ heterojunction architecture is commonly employed as a metal oxide photoanode for the water oxidation half-reaction. The energy level ordering results in a staggered heterojunction that can effectively separate photoexcited electrons into the WO_3_ layer towards the current collector and photoexcited holes into the BiVO_4_ layer towards the interface with the electrolyte. Chemical vapour deposition (CVD) is an upscalable technique for fabricating large-area thin films of a wide range of semiconductors with nanoscale control. The fluorine-doped tin oxide (FTO)-coated transparent conductive glass substrates used herein are mass-produced by the glass industry with atmospheric pressure CVD and so the entire photoelectrode could be produced in one production process on float glass panels. This work is a detailed study of the use of atmospheric pressure CVD to fully-fabricate high-performance BiVO_4_-coated WO_3_ nanostructures (500–2000 nm in length with 25–100 nm thick BiVO_4_ coatings) for photoelectrochemical (PEC) water splitting. Incident photon-to-current efficiency measurements were used to calculate optimal solar predicted photocurrents of 1.92 and 2.61 mA cm^−2^ (2.3% and 3.2% solar-to-hydrogen efficiency if coupled to a hypothetical photovoltaic providing 1.23 V) for WO_3_/BiVO_4_ heterojunction samples under front and back-illumination, respectively. The heterojunction showed more than additive improvements over the parent materials, with bare WO_3_ and BiVO_4_ samples showing 0.68 and 0.27 mA cm^−2^ and 0.50 and 0.87 mA cm^−2^ under front and back-illumination, respectively. Simulations of the current–voltage characteristics of tandem crystalline silicon photovoltaic modules coupled to the PEC devices were consistent with the solar predicted photocurrents. These promising results for BiVO_4_-coated WO_3_ nanoneedles fully-deposited by atmospheric pressure CVD enables future research into photoanodes amenable to large-area scale-up.

## Abstract 2

WO_3_/BiVO_4_ heterojunction layer space systematically explored using chemical vapour deposition synthesis to map and identify samples of highest performance for solar water splitting applications.

## Graphical abstract

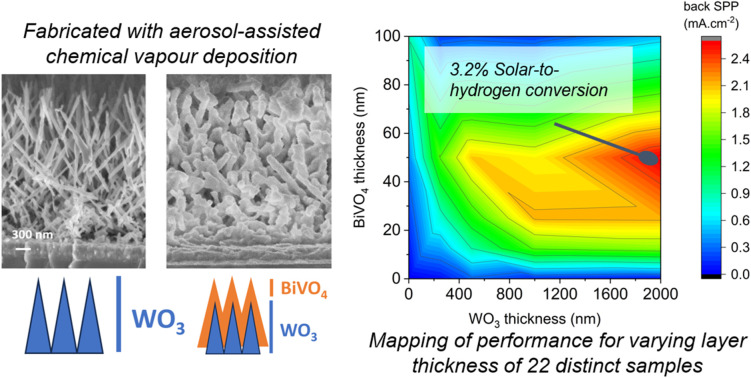

## 1. Introduction

Technologies that store renewable but intermittent solar energy as a chemical fuel will help society reduce its dependence on fossil fuel extraction. One such technology, photoelectrochemical (PEC) water splitting, uses sunlight to drive the reduction and oxidation of water into hydrogen fuel and oxygen gas at the surfaces of a (photo)cathode and a (photo)anode respectively.^1^ PEC water splitting, however, is still an immature technology, with both laboratory and prototype-scale research underway to improve efficiency, durability, and scalability.^2,3^ In contrast to how crystalline Si has become the archetypal low-cost photovoltaic solar cell,^4^ no single device design, light absorber nor catalytic material system has emerged as a defining example for efficient, low-cost and scalable PEC water splitting.^5^ The Solar Fuels Database^6^ collates data on reported PEC water splitting devices. The solar-to-hydrogen (STH) conversion efficiencies for PEC devices made using Si and III–V semiconductors have reached 19%,^7^ and the STH conversion efficiencies for devices made using lower-cost, earth-abundant metal oxides have reached ∼10%,^6^ although only for small-scale devices. Notably, a minimum of 10% is reported as a requirement for the overall performance of PEC water splitting to be comparable to PV-coupled electrolysis,^8^ with values as high as 20–26% targeted by the U.S. Department of Energy.^9^ Reaching these efficiencies with non-precious materials, whilst also demonstrating acceptable stability, scalability and cost targets, remains a significant challenge for the PEC water splitting research community.

Si and III–V semiconductor-based devices typically use light absorbers known to be effective in photovoltaic (PV) devices and then add protective passivation layers to prevent their corrosion and promote catalysis in an electrolyte.^10^ This method of adding complexity to a traditional PV device is, however, unlikely to be cost-effective compared to PV-coupled electrolysis,^8^ which does not have to mitigate the effects of having the electrolyte in close proximity to the light absorbers. Transition metal oxides, in contrast, have higher durability, lower cost, and can be more readily produced using scalable synthesis, and thus are the most commonly studied class of material for PEC water splitting.^2^ One limitation to the water splitting performance of metal oxides is their often wide band gaps that can restrict their ability to harvest solar energy.^11^ Nevertheless, using multiple distinct light absorbing layers in tandem in a type II heterojunction with staggered conduction and valence bands can enable wider light absorption and maintain efficient transfer of electrons and holes through the layered architecture.^12^ The bismuth vanadate-coated tungsten trioxide (WO_3_/BiVO_4_) photoanode is one such example of a tandem type II heterojunction.^13^ WO_3_ has been shown to have a long hole diffusion length (∼150 nm) and good electron mobility (∼12 cm^2^ V^−1^ s^−1^), but suffers from slow charge transfer at the semiconductor/electrolyte interface^14^ and can only absorb blue and ultraviolet light with its 2.7–2.8 eV bandgap energy.^15,16^ BiVO_4_, with its indirect bandgap of 2.4 eV,^17^ absorbs up to green light, but is hindered by electron–hole recombination and poor charge transport and water oxidation kinetics.^18^ Together, however, photocatalysts with a WO_3_/BiVO_4_ sputter-fabricated core–shell architecture, when coupled with a double-junction GaAs/InGaAsP photovoltaic cell, have demonstrated a record 8.1% STH conversion efficiency for BiVO_4_-based photoanode water splitting devices.^19^ This heterojunction configuration of light absorbers benefits from improved charge separation across the interfacial region,^20^ while the nanostructuring allows for a simultaneously thick BiVO_4_ layer along the length of the WO_3_ and a radially thin BiVO_4_ layer that mitigates for the poor charge mobility through BiVO_4_.^21,22^ Metal oxide photocathodes, such as those based on Cu_2_O, have been demonstrated with up to approximately 7.6 mA cm^−2^ or 9.3% STH efficiency when measured at 0 V *vs.* RHE in a half-cell configuration.^23^ The challenge to using photocathodes is that they are typically sensitive to photocorrosion and so require complex capping layers to function. The remainder of this work is focused on photoanodes.

Future commercial PEC water splitting facilities may require photoabsorbers with large areas on the order of centimetres to meters in dimensions.^24^ The WO_3_/BiVO_4_ heterojunction photoabsorber literature to date have, however, primarily employed fabrication techniques such as sputtering under vacuum,^25^ or electrochemical^26^ and spin-coating methods^27,28^ that may be difficult to apply to large-area coating. Along with spray pyrolysis,^29,30^ chemical vapour deposition (CVD) is a route to making metal oxide thin films that can be scaled to large areas and has been demonstrated to be capable of producing a wide range of inorganic photoabsorbers.^2^ Seminal works on water splitting photocatalysts fabricated by CVD include the vapour transport deposition of BiVO_4_,^31^ aerosol-assisted chemical vapour deposition (AACVD) of BiVO_4_,^32^ and demonstration and optimization of WO_3_ nanoneedles by AACVD.^33,34^

This work demonstrates, to our knowledge, the first nanostructured WO_3_/BiVO_4_ heterojunction photoanode where both components are deposited sequentially by AACVD. BiVO_4_ is conformally coated onto WO_3_ nanoneedles on FTO-coated glass to form dense coatings 25–100 nm thick surrounding micron-length, 50–100 nm diameter nanoneedles, as seen by scanning electron microscopy. The characteristic X-ray crystal diffraction, X-ray photoemission spectroscopy, and Raman spectroscopy features for WO_3_ and BiVO_4_ are unchanged when the heterojunction is formed, indicating that each component retains its physical integrity, and that the heterojunction has a distinct interface. Additionally, we also investigate the importance of preventing the aging of the vanadium precursor solution (vanadium(iii) acetylacetonate in acetone and methanol), where tests were conducted to help elucidate the mechanism for CVD deposition of BiVO_4_, adding to the emerging body of knowledge in this field.^35^ The WO_3_/BiVO_4_ heterojunction photoanode showed significantly enhanced activity for water oxidation and a superior calculated solar predicted photocurrent (SPP) compared to single component WO_3_ and BiVO_4_ photoanodes, validating the enhanced absorptance properties of the heterojunctions as measured by UV-visible spectroscopy. Modelling of a PV-PEC hybrid device, carried out herein, where a back-illuminated WO_3_/BiVO_4_ heterojunction is coupled to dual crystalline Si PVs placed electrically in series and optically in tandem, estimates that our photoanodes can achieve an unassisted STH of up to 3.4%. Overall, we demonstrate that AACVD can fabricate WO_3_/BiVO_4_ heterojunction photoanodes with promising performance for large-scale device applications.

## 2. Results & discussion

### 2.1 Synthesis of the WO_3_ and BiVO_4_ photoanodes

A wide range of WO_3_ nanoneedles (NN), BiVO_4_, and BiVO_4_-coated WO_3_ samples were fabricated in triplicate and are listed in Table 1 according to their deposition volumes for aerosol-assisted chemical vapour deposition and approximate layer thicknesses determined by side-on scanning electron microscopy (SEM). Fig. S1† shows representative photographs of the post-annealed FTO-glass substrates coated with WO_3_ NN, BiVO_4_, and BiVO_4_-coated WO_3_ along with a photograph of the CVD reactor that can deposit samples up to 16 cm × 5 cm in surface area. The FTO-coated substrates used herein are mass-produced by the glass industry with atmospheric pressure CVD^36^ and so there is the possibility for the entire photoelectrode to be produced in one production process on float glass panels. WO_3_ films change from a deep blue (sub-stoichiometric WO_3−*x*_) to white colour (near stoichiometric WO_3_) when annealed in air. Annealing removes trace contaminants, oxidizes the film, and a two-hour annealing time retains optimal ∼2% levels of oxygen vacancies^37^ enhancing the photoactivity of the WO_3_ photoanodes.^38,39^ Annealing BiVO_4_ films ensures complete conversion of the films to the more photoactive monoclinic crystal phase^40^ and oxidation to yellow BiVO_4_ with V in the 5+ oxidation state. The rough surface of the WO_3_ NN scatter light and cause the films to appear opaque. The BiVO_4_ on FTO is flat in comparison and appears transparent, while on WO_3_, the BiVO_4_ coats the NN conformally, so the final heterojunction sample also appears opaque.

**Table 1:** Combinations of WO_3_ and BiVO_4_ fabricated and studied in triplicate. V (mL) is the precursor volume (with concentrations of 11.4 mM for WO_3_ and 5 mM for BiVO_4_ precursor solutions) used to produce the layers, *L* (nm) is the approximate length of the WO_3_ NN produced, and *t* (nm) is the approximate thickness of the BiVO_4_ coating on WO_3_ NN. Tick/cross symbols are used to designate samples that were/were not synthesized, with a total of 22 distinct samples studied herein

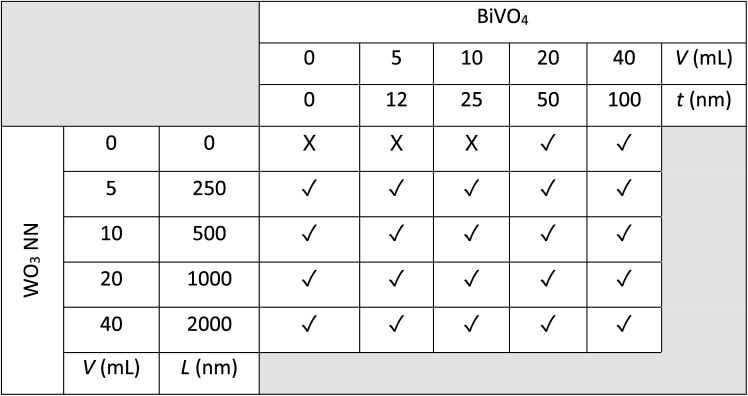

#### 2.1.1 Scanning electron microscopy (SEM) imaging

**Fig. 1:**
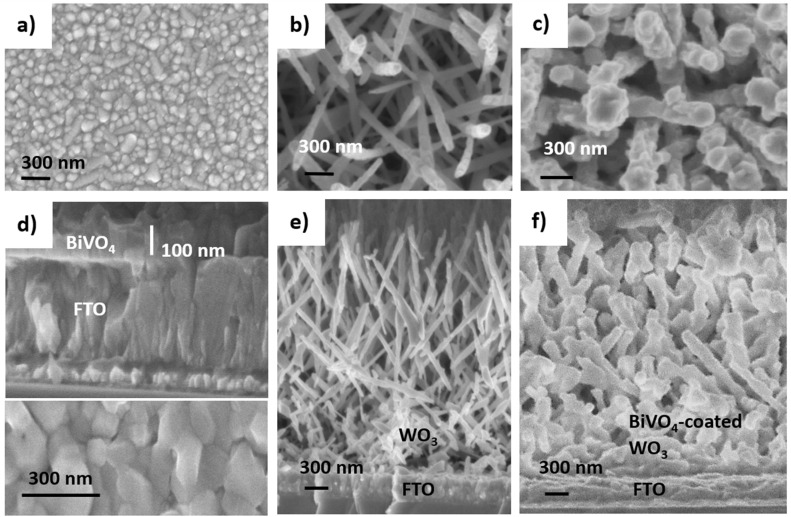
Exemplar SEM images of (a) FTO substrate, (b) top view of WO_3_ NN, (c) top view of WO_3_ NN coated with 100 nm thick BiVO_4_, (d) (top): side-on view of 100 nm thick BiVO_4_ on FTO, (bottom): top-down view of BiVO_4_, (e) side-on view of 4000 nm long WO_3_ NN on FTO and (f) side-on view of 100 nm thick BiVO_4_ coated on 4000 nm long WO_3_ NN on FTO.

SEM imaging (Fig. 1) was conducted to show the morphology of representative heterojunction films. Thicker films made from 80 mL of WO_3_ precursor (11.43 mM W(CO)_6_ dissolved in 3 : 1 acetone and methanol) and 40 mL of BiVO_4_ precursor (5 mM triphenylbismuth and vanadium(iii) acetylacetonate dissolved in 3 : 1 acetone and methanol) were fabricated and measured to more easily image the distinct layers on top of the FTO-coated glass substrate, Fig. 1a, in contrast to the thinner layers described in Table 1. Fig. 1b shows the top-down view of WO_3_ NN, which forms as sharp needles (50–100 nm in diameter) growing predominantly upwards in a range of angles. Fig. 1c shows the top-down view of WO_3_ NN that have become more globular upon coating with BiVO_4_. The final diameter of the BiVO_4_-coated WO_3_ needles are between 250 and 300 nm, corresponding to a radial coating of about 100 nm, herein referred to as the BiVO_4_ thickness. BiVO_4_ on bare FTO-coated glass, shown from side-on and top-down views in Fig. 1d, coats as a thicker film than on WO_3_ NN; 10 mL precursor solution is used to make the coating in Fig. 1d compared to 40 mL for the coatings in Fig. 1c and f. Fig. 1e and f, which are the side-on images of WO_3_ NN and NN coated with BiVO_4_, on FTO. The majority of NN are pointing upwards from the FTO. Notably, the AACVD coating process for the BiVO_4_ layer fully penetrates to the bottom of the host nanostructure, while preserving the needle-structure of the WO_3_.

### 2.2 Hypothesis for the mechanism of BiVO_4_ deposition

Understanding the mechanism for CVD deposition of the ternary BiVO_4_ is important for ensuring high quality depositions. The deposition is more complex than for binary oxides like WO_3_,^41^ and has not previously been discussed in the literature.^31,32,42,43^ In this work, we use thermogravimetric analysis (TGA) and UV-vis of the precursor solutions along with a temperature-dependent X-ray diffraction study to help determine the decomposition dynamics of the BiVO_4_ precursors. The findings enable us to formulate the hypothesis that differences in activation energy between bismuth and vanadium metal–organic precursors leads to controlled product stoichiometry with changing deposition temperature.

**Fig. 2:**
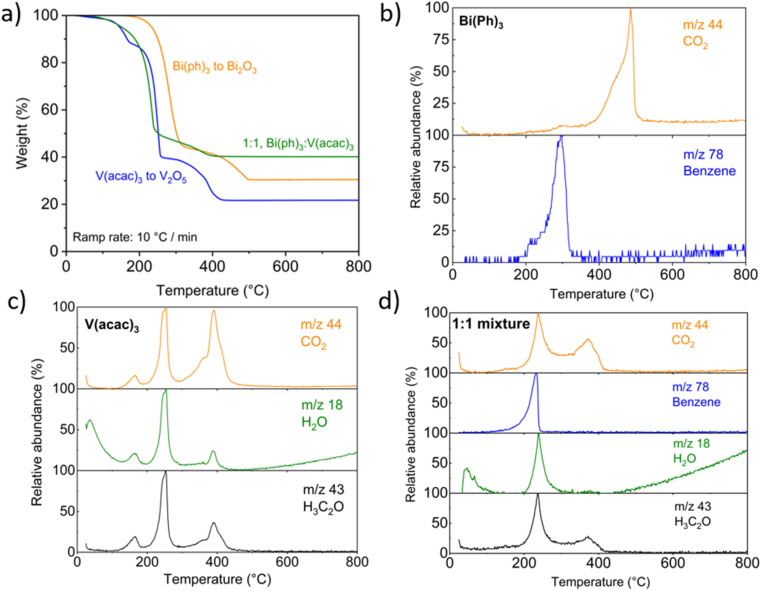
TGA-mass spectrometry (MS) of the precursors used in the AACVD synthesis of BiVO_4_ (a) weight loss % as a function of temperature, and mass spectrometry of volatile materials for (b) Bi(Ph)_3_, (c) V(acac)_3_, and (d) 1 : 1 mixture of Bi(Ph)_3_ and V(acac)_3_.

TGA in air coupled with mass spectrometry was conducted on the neat precursor powders, triphenylbismuth, Bi(Ph)_3_, and vanadium(iii) acetylacetonate, V(acac)_3_, to investigate their decomposition at the temperatures used in our CVD reaction. Bi(Ph)_3_ is expected to decompose to Bi_2_O_3_, V(acac)_3_ to V_2_O_5_ and the 1 : 1 mixture to BiVO_4_ as these are the common fully oxidated forms. In Fig. 2a the TGA profiles show that Bi(Ph)_3_ appears to lose mass primarily between 200–300 °C and then 400–500 °C. The V(acac)_3_ loses mass during three temperature ranges between 150–200 °C, 200–250 °C, and 300–400 °C. A 1 : 1 mixture of Bi(Ph)_3_ and V(acac)_3_ also appears to lose mass in two major, albeit prolonged temperature ranges, between 150–250 °C and then 250–400 °C. The final residual mass of 22% for V(acac)_3_ decomposition to V_2_O_5_ was close to the expected value of 26%, while for Bi(Ph)_3_ decomposition to Bi_2_O_3_, the 30% final residual mass was less than the expected value of 53%, which may be caused by partial sublimation of Bi(Ph)_3_,^44^ although any Bi-containing species were too heavy to be measured in the TGA-MS. Importantly, the final residual mass for the decomposition of the 1 : 1 mixture to BiVO_4_ of 40% matches the expected value of 41%.

The MS of Bi(Ph)_3_ as a function of temperature, shown in Fig. 2b, shows a distinct release of benzene attributed to the release of the phenyl ligand between 200 and 300 °C, which corresponds to the first mass loss seen in the TGA. There is a brief pause in mass change before a second mass loss at 400 °C, corresponding to the release of CO_2_ until 500 °C, which may be attributed to the combustion of stray phenyl remaining in the material. In Fig. 2c, the MS of V(acac)_3_ shows an initial loss of H_2_O up to 100 °C which is likely surface adsorbed water from storage of the material in air. There are then three distinct peaks at 150 °C, 250 °C and 400 °C where characteristic fragments of the acetylacetone (*m*/*z* 43), water and CO_2_ are released, matching the temperatures for mass losses in the TGA. The area of the MS peaks also corresponds with the relative mass losses. In Fig. 2d, MS of the 1 : 1 mixture of Bi(Ph)_3_ and V(acac)_3_ shows all expected peaks from the individual materials with lower resolution, suggesting that decay of the solid-state mixture is generally a combination of the constituent individual compounds, and not from other (mixed-metal) intermediates. The V(acac)_3_ precursor loses mass, and the corresponding products are observed by mass spectroscopy at approximately 50 °C lower compared to the Bi(Ph)_3_ precursor, indicating that V(acac)_3_ has a lower activation energy for decomposition than Bi(Ph)_3_.^45^

The choice of precursors should have an influence on the reaction mechanism for the deposition of the ternary metal oxide. An alternative commonly studied vanadium precursor for CVD is vanadyl(iv) acetylacetonate, VO(acac)_2_.^46^ VO(acac)_2_ has been reported with TGA to fully decompose by about 300 °C,^47^ approximately 100 °C lower than for the complete decomposition of V(acac)_3_. If VO(acac)_2_ is used in AACVD with Bi(Ph)_3_, there may then be a wider range of temperatures in which the two precursors have differing levels of decomposition. Use of VO(acac)_2_ with Bi(Ph)_3_ should then be avoided when stoichiometric BiVO_4_ is desired. According to UV-vis studies shown in Fig. S2,† V(acac)_3_ decomposes and oxidises into VO(acac)_2_ under atmospheric conditions over time when dissolved in 3 : 1 acetone : methanol. The oxidation process occurs rapidly over the first few hours and is followed by further oxidation to generate primarily V(v) species over the course of two days. Therefore, we postulate that conducting AACVD using solutions containing V(acac)_3_ aged for even only a few hours, may also have the same effect of using the VO(acac)_2_ precursor directly, and, therefore, recommend the immediate use of V(acac)_3_ stored under inert gases upon dissolution in the 3 : 1 acetone : methanol solution for CVD synthesis (where herein, depositions were conducted within ∼15 min of dissolution).

**Fig. 3:**
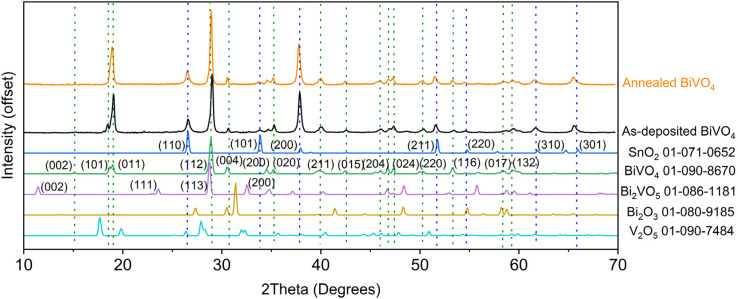
XRD patterns for BiVO_4_ films grown on FTO-coated glass by AACVD before annealing (as deposited) and after annealing at 500 °C for 2 hours in air. Data is plot alongside PDF card references patterns for SnO_2_ (*i.e.* the FTO substrate), BiVO_4_, Bi_2_VO_5_, Bi_2_O_3_ and V_2_O_5_.

Flat BiVO_4_ films prepared on FTO-coated glass at 400 °C show the XRD patterns for stoichiometric BiVO_4_ (Fig. 3). The as-deposited sample shows the (002) (101) (011) (112) (004) (200) (020) (211) (015) (204) (024) (220) (116) (017) & (132) peaks corresponding to polycrystalline BiVO_4_ (monoclinic *I*112/*b*^15^*a* = 5.1907 Å, *b* = 5.0912 Å, *c* = 11.6941 Å; *α* = *β* = 90°, *γ* = 90.360°; PDF no. 01-090-8670) along with the (110) (101) (200) (211) (220) (310) & (301) diffraction peaks for the FTO substrate (tetragonal *P*4_2_/*mnm* (136); *a* = *b* = 4.7380 Å, *c* = 3.1865 Å; *α* = *β* = *γ* = 90°; PDF no. 01-071-0652). The effect of annealing the BiVO_4_ serves to eliminate the peak corresponding to the (101) crystalline plane, leaving only the (011) peak in the region near 19°. Peaks corresponding to Bi_2_VO_5_ (PDF no. 01-086-1181), Bi_2_O_3_ (PDF no. 01-080-9185) and V_2_O_5_ (PDF no. 01-090-7484) are not evident before or after annealing, indicating that the materials are deposited in a stoichiometric ratio and do not segregate into phases of the binary metal oxides.

Next, flat BiVO_4_ films were grown by CVD on FTO-coated glass for reactor temperatures between 250 and 450 °C. In these experiments the vanadium precursor was aged for one month in the solid state and therefore a minute amount of Bi_2_VO_5_ was expected. Fig. S3† shows the XRD patterns for these films before and after annealing at 500 °C in air for two hours. For relatively low temperatures, at 250 °C neither precursor has begun to significantly decompose, so both species deposit on the substrate as amorphous solid solutions and the final film after annealing reflects the starting precursor stoichiometry. At relatively high deposition temperatures, 400 °C and 450 °C, both precursors rapidly decompose and crystallise together as-deposited, resulting in annealed films that also reflect the starting precursor stoichiometry. For moderate deposition temperatures at 300 °C and 350 °C, the V(acac)_3_ decomposes to a greater extent compared to the Bi(Ph)_3_. Under these conditions, the lighter V(acac)_3_ derivatives may be re-evaporated into the passing aerosol stream, increasing the relative amount of Bi in the film, with an additional Bi_2_VO_5_ phase identified, evidenced by the relatively large and broad peak corresponding to the Bi_2_VO_5_ (002) plane present after annealing.

These results suggest that differences in activation energy between the bismuth and vanadium metal–organic precursors allows for the control of BiVO_4_ stoichiometry with changing deposition temperature, with an optimal deposition temperature of 400 °C for fabricating BiVO_4_ for no observable sub-stoichiometric phases.

### 2.3 Chemical analysis of the WO_3_ and BiVO_4_ photoanodes

#### 2.3.1 X-ray diffraction (XRD) and Raman spectroscopy

**Fig. 4:**
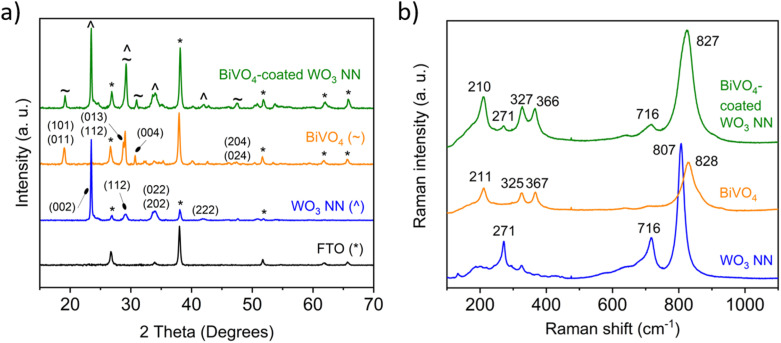
(a) XRD patterns and (b) Raman spectra for 1000 nm long WO_3_ nanoneedles, BiVO_4_ and 50 nm thick BiVO_4_ coated on WO_3_ nanoneedles corresponding to monoclinic WO_3_ (ref. 34, 48 and 49) and monoclinic BiVO_4_.^50,51^

Fig. 4a shows XRD patterns for representative samples of nanostructured WO_3_, BiVO_4_, and BiVO_4_-coated WO_3_, grown on FTO-coated glass, with their characteristic Miller indices labelled.^48,49^ The nanostructured WO_3_ is preferentially orientated in the (002) plane and a single peak near 23° dominates, corresponding to monoclinic WO_3_ (*P*12_1_/*n*1;^14^*a* = 7.3271 Å, *b* = 7.5644 Å, *c* = 7.7274 Å; *α* = *γ* = 90°, *β* = 90.488°).^34^ Peaks associated with the FTO substrate are indicated with asterisks (*). The BiVO_4_-coated WO_3_ samples have XRD peaks corresponding to both the polycrystalline monoclinic BiVO_4_ (ref. 50 and 51) and WO_3_ sample patterns. The peaks associated with nanostructured WO_3_ are preserved, even after the deposition and annealing of BiVO_4_, indicating the formation of a sharp heterojunction between the two materials.

The Raman spectra shown in Fig. 4b show characteristic peaks matching literature spectra of WO_3_ (ref. 52 and 53) and BiVO_4_, with the peak at 828 cm^−1^ corresponding to the A_g_ symmetric vibrations of V–O bonds, the peaks at 366 and 327 cm^−1^ corresponding to the B_g_ asymmetric and A_g_ symmetric modes of VO_4_^3−^ respectively^54^ and the peak at 211 cm^−1^ being an external mode.^55,56^ For the heterojunction spectrum, the widened peak seen at 827 cm^−1^ is likely due to the combination of the A_g_ peak at 807 cm^−1^ for WO_3_ and the A_g_ peak at 828 cm^−1^ for BiVO_4_.^57^ The superimposed peaks of the BiVO_4_-coated WO_3_, therefore, suggest that distinct phases of BiVO_4_ and WO_3_ form in the heterostructure, and that a distinct interface is formed.

#### 2.3.2 X-ray photoelectron spectroscopy (XPS)

Exemplar flat samples of approximately 200 nm thick WO_3_, BiVO_4_, and BiVO_4_-coated WO_3_ on FTO-coated glass were measured by XPS to obtain information about their work function and stoichiometry. Average work function values were measured to be 4.3 and 4.5 eV below vacuum for WO_3_ and BiVO_4_ respectively, detailed in Table S1.† These values both shift to 4.8 eV upon surface cleaning with an argon ion-beam etch (evidenced by removal of the adventitious C 1s peak). The heterojunction BiVO_4_-coated WO_3_ film has a similar work function of 4.5 eV before and 4.6 eV after etching.

Peak fitting of high-resolution XPS spectra confirm near stoichiometric values for WO_3_ and BiVO_4_. Atomic% values of 25% for W^6+^, 2% for W^5+^, and 73% for O^2−^ were observed and is commensurate with the expected stoichiometry of WO_3_ with approximately 2% oxygen vacancies. 14% Bi^3+^, 12% V^5+^, 6% V^4+^, 1% V^3+^, and 67% O^2−^ were the at% values observed for BiVO_4_. The proportion of oxygen matches the expected stoichiometry, while the total amount of vanadium is similar to the bismuth. The mixture of vanadium reduced states may be induced by X-ray reduction during the XPS measurement. Survey scans and high-resolution peak fitting with elemental quantification are shown in Fig. S4 for WO_3_ and S5 for BiVO_4_.† The peak fit parameters are also detailed in Tables S2 and S3† for WO_3_ and BiVO_4_ respectively.

### 2.4 Optical characterisation of the WO_3_ and BiVO_4_ photoanodes

**Fig. 5:**
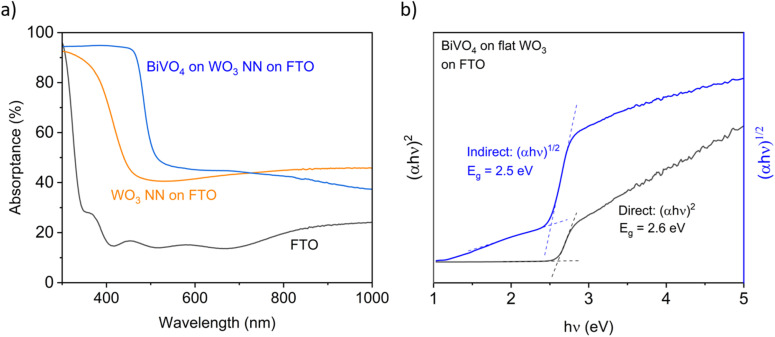
(a) UV-vis absorptance for 2000 nm thick WO_3_ NN and 100 nm BiVO_4_-coated NN WO_3_ compared to FTO-coated glass and (b) Tauc plots derived from measurements of transmittance and total reflectance of 400 nm planar BiVO_4_ on 100 nm planar WO_3_ on FTO-coated glass.

Fig. 5a compares the absorptance spectra calculated from measured transmittance and total reflectance spectra (Fig. S6a and S6b†) of representative photoanode films of 2000 nm thick WO_3_ nanoneedles (NN) and 100 nm of BiVO_4_ conformally coated on 2000 nm thick WO_3_ NN on FTO-coated glass. As each layer is added, the absorption onset of the samples shifts from 350 nm for FTO-coated glass to 450 nm for WO_3_-coated FTO, and finally to 510 nm for BiVO_4_-coated WO_3_ on FTO. These values are consistent with the expected bandgaps for SnO_2_, WO_3_ and BiVO_4_ of 3.6,^58^ 2.8 and 2.4 eV,^11^ respectively. The overall optical properties of the layered materials are dominated by the absorptance of the constituent material with the smallest bandgap energy. For a more precise determination of the bandgap, a less scattering exemplar film was used, which consisted of a 400 nm thick BiVO_4_ layer coated onto a 100 nm thick flat (not nanostructured) WO_3_ underlayer. Flat WO_3_ is fabricated with a similar CVD procedure as for nanostructured WO_3_ using a lower substrate temperature.^34^ The measured transmittance and total reflectance spectra of this film was used to produce a Tauc plot, shown in Fig. 5b, which revealed a 2.5 eV indirect transition and 2.6 eV direct transition for the BiVO_4_ layer. The transmittance and reflectance spectra of the flat films are compared to the nanostructured films in Fig. S6a and S6b† showing the WO_3_ NN are less transmitting and more reflecting than flat WO_3_, but samples of BiVO_4_ coated on flat and NN WO_3_ have similar properties. Fig. S7a and S7b† show Tauc plots for WO_3_ and BiVO_4_ grown directly on FTO, indicating indirect bandgaps of approximately 2.8 eV and 2.5 eV, respectively.

#### 2.4.1 Analysis of light attenuation through the photoanode layers

**Fig. 6:**
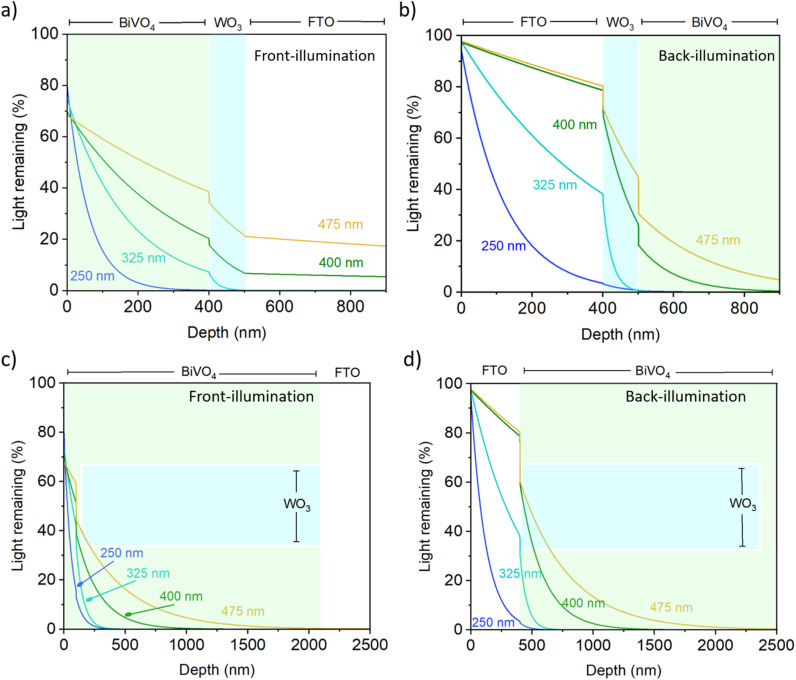
Calculated optical propagation of light through example heterojunction structures. Planar FTO/WO_3_/BiVO_4_ heterojunctions, with each respective layer being 320, 100 and 400 nm thick, for the case of (a) front illumination and (b) back illumination, and nanostructured FTO/WO_3_/BiVO_4_ heterojunctions with the FTO layer being 320 nm thick, coated with WO_3_ NN that are 100 nm wide and 2000 nm long, that are conformally coated with a 100 nm layer of BiVO_4_, for the case of (c) front illumination and (d) back illumination. Pastelle shading represents the layers of the heterojunction light is passing through; colourless = FTO, blue = WO_3_, green = BiVO_4_. Calculations were carried out for 250, 325, 400 and 475 nm light.

Fig. 6 shows calculated light absorption through exemplar planar and nanostructured heterojunction films, modelled using data from UV-visible spectroscopy of films with known layer thicknesses. Optical losses, due to the reflection of light, are accounted for at each interface. For this analysis, the level of reflection (both specular and diffuse) that may occur at each interface are considered, with the nanostructured samples showing a higher degree of scattering (which can be seen with higher reflection losses between each interface in Fig. 6). The results illustrate how nanostructured samples can better harvest light at the band edge of the material compared with the flat heterojunction. First-order reflection at the interfaces of each layer are considered as losses, which is a simplification, but a conservative assumption at that. Also, light scattering would be expected for realistic nanostructured samples, but this effect would further increase absorption due to the additional chances for absorption and so the key takeaways are reinforced.

The optical behaviour of a planar WO_3_/BiVO_4_ heterojunction system are shown in Fig. 6a and b, for back and front illumination, respectively. For such a system to be functional for water splitting, the planar layers cannot be too thick, or else both hole diffusion to the BiVO_4_ surface to drive water oxidation reactions and electron diffusion out of BiVO_4_ into WO_3_ would be inhibited. Thus, herein, we modelled the case for a 100 nm thick WO_3_ layer and a 400 nm thick BiVO_4_ layer. Under front illumination (Fig. 6a), UV light is effectively harvested by the BiVO_4_ layer, with ∼80% of 250 nm and ∼70% of 325 nm absorbed. Blue light is also strongly absorbed, with only ∼20% of 400 nm and ∼40% of 475 nm light remaining. Much of this light is then absorbed by the WO_3_ layer. Under back illumination (Fig. 6b), UV light of 250 nm is almost all absorbed by the FTO layer, and therefore cannot be harvested by the heterojunction. Similarly, ∼60% of UV light of 325 nm is absorbed by the FTO layer. This is then strongly absorbed by the proceeding WO_3_ layer. For the cases of 400 and 475 nm light, these are not significantly attenuated by the FTO layer. About 40% of 400 nm light and 20% of 475 nm light is absorbed by the WO_3_ layer, with only ∼20% of the remaining 400 and 475 nm light absorbed upon reaching the BiVO_4_ layer. Overall, back illumination of the planar heterojunction shows that UV light (*i.e.* 250 and 325 nm) cannot be harvested effectively due to parasitic absorption by the FTO layer, and that light near the band edge of BiVO_4_ (*i.e.* 475 nm) is not effectively harvested either.

For the case of a nanostructured heterojunction WO_3_/BiVO_4_ system, the nanostructured WO_3_ layer can decouple the limited electron and hole diffusion of the conformally coated BiVO_4_. Moreover, given the excellent electron transport found in WO_3_, one can use a microns-thick nanostructured layer. Thus, herein, we modelled the case for a nanostructured WO_3_ layer with rods 100 nm wide and 2000 nm long that are coated with a 100 nm conformal BiVO_4_ layer, like what we have produced experimentally herein (Fig. 1). A cross-section is modelled that transects the middle of the nanorod, where light encounters the WO_3_ layer one-third of the time and the BiVO_4_ layer two-thirds of the time. For the case of front illumination (Fig. 6c), all light from 250 to 475 nm is harvested within the WO_3_/BiVO_4_ nanostructure, which results in optimal charge carrier production. Under back illumination (Fig. 6d), significant absorption within the FTO layer is seen for 250 nm and 325 nm light, just as for the planar heterojunction (Fig. 6c). Different behaviour is, however, seen for longer wavelengths, with all 400 and 475 nm light absorbed by the nanostructured architecture because of the increased length of the absorber. The BiVO_4_ layer in the nanostructured architecture is more capable of harvesting light, particularly wavelengths at the band edge of the material where a more significant portion of the solar spectrum resides. The micron-scale distance that light must travel through the BiVO_4_ in the nanostructured heterojunction results in all the light being absorbed, whereas residual light is transmitted through the flat heterojunctions. Under back illumination, exciton electron–hole pairs are formed much closer to the FTO electron collector than for photoanodes under front illumination.

### 2.5 Photoelectrochemical (PEC) experiments

#### 2.5.1 Current voltage (*J*–*V*) investigation

**Fig. 7:**
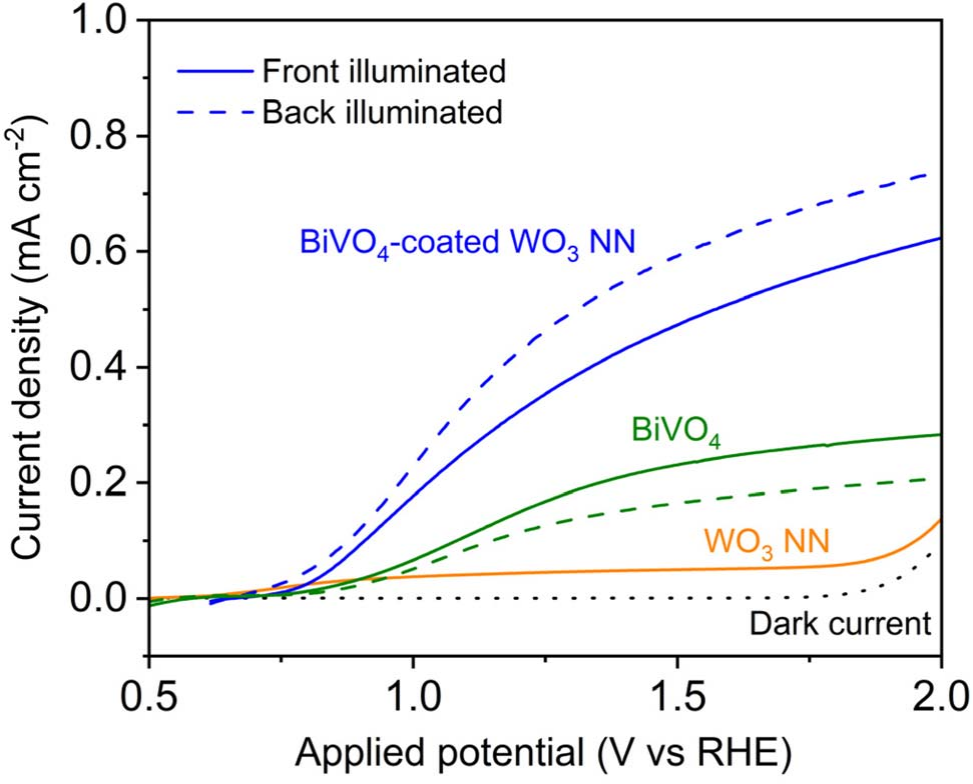
*J*–*V* curves for 1000 nm thick WO_3_ NN (pH 1.1, 0.1 M H_2_SO_4_ (aq.)), 200 nm thick BiVO_4_ and 50 nm thick BiVO_4_-coated 1000 nm thick WO_3_ NN (pH 7, 0.1 M K_2_HPO_4_/KH_2_PO_4_ (aq.)) on TEC15 FTO-coated glass. Scan rate: 10 mV s^−1^, illuminated with a 75 W Xe lamp attenuated with a neutral density filter at approximately 1 sun intensity. Solid lines represent front illumination, and dashed lines represent back illumination.

Fig. 7 compares representative *J*–*V* curves of PEC modules using WO_3_ NN or BiVO_4_ as the photoanode with the *J*–*V* curve of the BiVO_4_-coated WO_3_ heterojunction photoanode under front and back illumination. The WO_3_ NN investigated are 1000 nm thick, and the BiVO_4_ is 200 nm thick when deposited on FTO and 50 nm thick when coated on WO_3_. The heterojunction photocurrent density is significantly increased compared to photoanodes made with a single constituent material, which evidences the effect of improved charge separation across the heterojunction interface. The photocurrents of 0.35 and 0.45 mA cm^−2^ for the heterojunction at an applied potential of 1.23 V *vs.* RHE under front and back-illumination respectively are significantly improved from the 0.16 and 0.12 mA cm^−2^ for BiVO_4_ under front and back illumination and 0.05 mA cm^−2^ for WO_3_ under front illumination. The low photocurrent for the WO_3_ photoanode is attributed to its large bandgap and limited visible light absorption. Furthermore, the white light of the 75 W xenon lamp used, although able to provide overall illumination matching 1 sun intensity, has discrepancies in its intensity of light with shorter wavelengths, shown in Fig. S8,† compared to the AM1.5G solar spectrum. This limitation reduces the apparent photocurrent measured using the white light, but there are significant cost benefits to using a 75 W xenon lamp. A spectrally matching solar simulator requires a 500 W or higher lamp coupled to air mass filters with a lifetime of only 1000 hours compared to 3000 hours for 75 W lamps.^59^ Incident photon conversion efficiency measurements using monochromated light fully mitigate this limitation and allow for direct comparison with the AM1.5G solar spectrum.

Current density losses along the length of the nanostructures and mass transport losses arising from the increased distance that reactants and products travel between the electrode surface and electrolyte bulk may limit the ultimate photocurrents. It was previously shown by Kafizas *et al.*, however, that nanostructured WO_3_ had significantly increased photoanode performance compared to flat WO_3_.^34^ This result was attributed to the full utilisation of photon penetration depth that increases the light absorption through the length of the needles.^34^ The increase in surface area likely also contributes to mitigating current density and mass transport losses in highly crystalline, annealed WO_3_ nanostructures where oxygen vacancy defects are minimized to improve electron transport.^60^

#### 2.5.2 Incident photon conversion efficiency (IPCE)

**Fig. 8:**
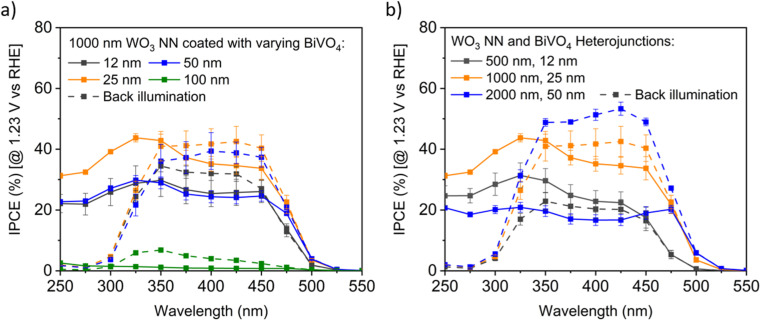
Exemplar IPCE profiles for BiVO_4_-coated WO_3_ on FTO, measured at pH 7 in 0.1 M KH_2_PO_4_/K_2_HPO_4_ (aq.) buffer electrolyte under front and back illumination (dashed lines), with 95% confidence intervals for triplicate measurements (a) varying BiVO_4_ on 1000 nm long WO_3_ nanoneedles, and (b) constant ratio of varying BiVO_4_ and WO_3_ nanoneedle thicknesses.

The IPCE profiles of 66 distinct samples (triplicates of 22 recipes) with differing WO_3_ and BiVO_4_ layer thickness, as described in Table 1, were measured at 1.23 V *vs.* RHE using monochromatic light from 250 to 550 nm. IPCE profiles from 7 of these samples are plotted in Fig. 8 to show trends in performance. Fig. 8a shows the IPCE profiles for various thickness of BiVO_4_ coatings on 1000 nm long WO_3_ NN. These samples of WO_3_ are best coupled with a 25 nm thick BiVO_4_ coating for optimal IPCE under both front and back illumination. In general, back illumination outperformed front illumination at longer wavelengths. At short wavelengths, back illumination is hindered by absorption from the FTO-coated glass of the photoanode, although the solar spectrum will contain limited photons with wavelengths shorter than 300 nm.^61^

Fig. 8b compares the IPCE profiles for samples with an aspect ratio of NN to BiVO_4_ thickness of 40 : 1. The thinnest samples with 500 nm long WO_3_ NN show the least IPCE under back illumination, the samples with 1000 nm long WO_3_ NN showed moderate efficiency for both front and back illumination, and the samples with 2000 nm long WO_3_ NN demonstrated the highest efficiency under back illumination. The difference in the photocurrents of the front and back-illuminated photoanodes for the thickest photoanode is likely due to more facile extraction of excited majority carrier electrons away into the circuit when the excitons are formed close to the substrate compared to when they are formed near the photoanode surface under front illumination. The optimal BiVO_4_-coated WO_3_ nanoneedle photoanodes show excellent visible light photoactivity with up to approximately 50% IPCE for visible light with wavelengths up to 450 nm.

#### 2.5.3 Photoanode water splitting stability

**Fig. 9:**
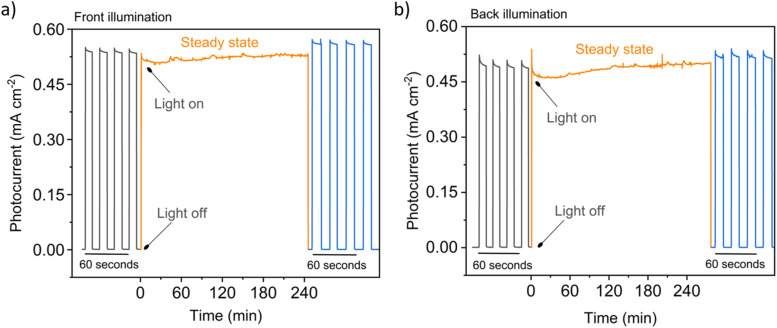
Chronoamperometry stability measurements of a representative 500 nm long WO_3_/50 nm thick BiVO_4_ heterojunction photoanode illuminated with a 75 W Xe lamp attenuated with a neutral density filter at approximately 1 sun intensity under (a) front and (b) back illumination. Electrolyte: 0.1 M, pH 7 KH_2_PO_4_/K_2_HPO_4_ (aq.), 1.23 V *vs.* RHE. *X*-axis scale varies as indicated.

The stability of a representative nanostructured WO_3_/BiVO_4_ photoanode was tested under illumination and 1.23 V *vs.* RHE applied potential. Fig. 9a and b show chronoamperometry measurements over four hours for front and back illumination, respectively. A chopped light measurement was taken (light on & off, in 10 second intervals) before and after the stability measurements and are shown on magnified timescales. Both measurements show stable photocurrents over four hours, which is commensurate with other studies of long-term BiVO_4_ (ref. 60) and BiVO_4_-based heterojunction^62^ photoelectrocatalysis, but further tests lasting hundreds of hours will be needed as evidence of commercially-applicable photocurrent stability. SEM imaging of a heterojunction sample after several hours of chronoamperometric and cyclic voltametric testing are depicted in Fig. S9† and show that the length of the nanoneedles remains stable. The needles are still coated in BiVO_4_, although there appears to be changes in shape of the surface of the material that may be a result of partial dissolution and rearrangement. The photocurrents recorded are lower than the solar predicted photocurrents discussed in the next section because of the mismatch between the spectrum of the xenon lamp and the solar spectrum, discussed earlier.

#### 2.5.4 Solar predicted photocurrent (SPP) for heterojunction devices

**Fig. 10:**
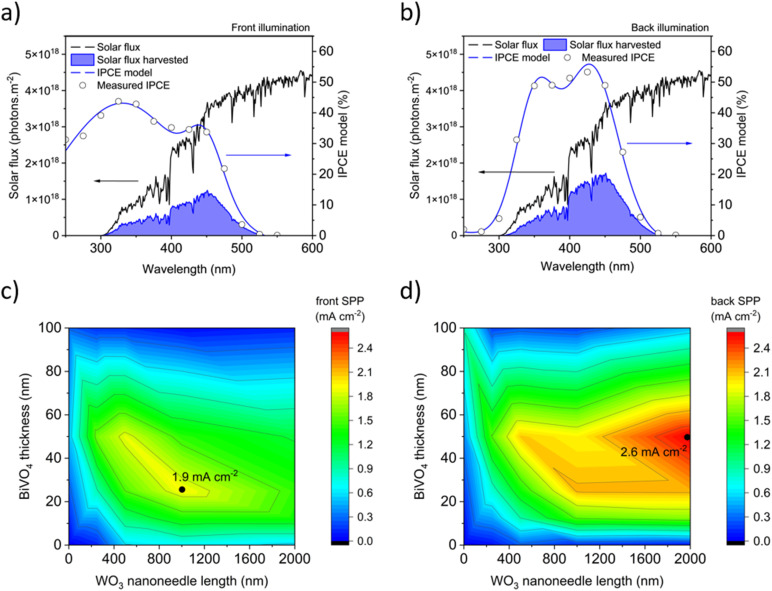
Calculations of solar predicted photocurrent (SPP) from the IPCE measured at 1.23 V *vs.* RHE for the nanostructured WO_3_/BiVO_4_ heterojunction samples showing the highest average performance under (a) front illumination (1000 nm thick WO_3_ NN coated with 25 nm of BiVO_4_) and (b) back illumination (2000 nm thick WO_3_ NN coated with 50 nm of BiVO_4_). Heat maps of the SPP for varying thicknesses of WO_3_ nanoneedles and BiVO_4_ in the heterojunction for the 22 unique samples examined for (c) front illumination and (d) back illumination.

The expected solar predicted photocurrent (SPP) of photoanodes illuminated by the AM1.5G solar spectrum is calculated by fitting a model to the IPCE curves to interpolate between the measured points. The conversion efficiency at each wavelength is then multiplied by the solar photon flux spectrum and integrated over all wavelengths. Fig. 10a and b show representative calculations of SPP for the optimal average IPCE profiles of 1000 nm thick WO_3_ NN coated with 25 nm of BiVO_4_ under front illumination and the 2000 nm thick WO_3_ NN coated with 50 nm of BiVO_4_ under back illumination, respectively. Most harvested photons have wavelengths between 400 and 450 nm due to the combination of high IPCE and high solar photon flux in this region. Fig. 10c and d show heatmaps of SPP created from the IPCE results of the 22 unique samples produced herein (Table 1) with varying WO_3_ nanoneedle and BiVO_4_ thickness for front and back illumination, respectively. The presence of BiVO_4_ is key to increasing the absorption of visible light of the WO_3_, and the heterojunction improves the SPP in all cases for moderate thicknesses of BiVO_4_. The average SPP for the front-illuminated devices peaks at 1.9 mA cm^−2^ for the samples with 1000 nm thick WO_3_ nanoneedles and a 25 nm coating of BiVO_4_, while the average SPP for the back-illuminated devices peaks at 2.6 mA cm^−2^ for the samples with 2000 nm thick WO_3_ nanoneedles and a 50 nm coating of BiVO_4_. If the applied potential (1.23 V *vs.* RHE) is provided from an additional solar-powered source placed optically in tandem (*e.g.* a PV), these values would equate to 2.3% and 3.2% solar-to-hydrogen (STH) conversion efficiency, respectively.

### 2.6 Model of a hypothetical PV-coupled PEC water splitting device

**Fig. 11:**
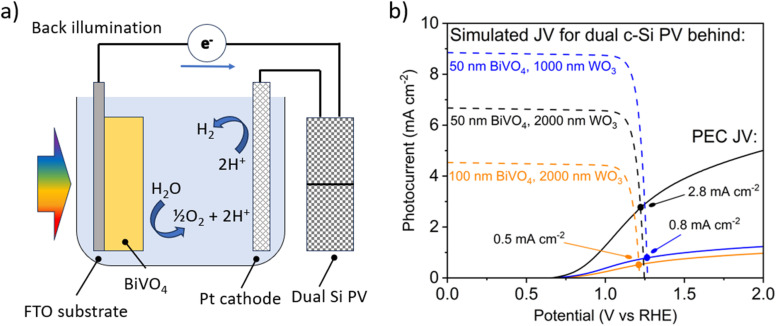
(a) Schematic of the hypothetical PV-PEC device with a dual c-Si PV electrically in series and optically in tandem with a back-illuminated PEC photoanode, (b) *J*–*V* curves for samples as indicated under back illumination as solid lines, and simulated *J*–*V* curves of corresponding dual c-Si PV modules behind the PEC module as dashed lines. Simulations and measured curves are calibrated to AM1.5G 1 sun illumination.

A hypothetical PV-coupled PEC water splitting (PV-PEC) device is arranged with a photoanode, metal cathode mesh, and silicon PV system optically in tandem as shown in Fig. 11a. The photoanode is illuminated from the backside to avoid being shaded by other device components such as the Pt cathode or electrolyte. The orientation of the cathode should be placed parallel to the photoanode for optimal mass transport of ions in solution and to reduce pH gradients in electrochemical set-ups.^30^ In our hypothetical device, the dual c-Si PV collects the transmitted light after passing through the whole PEC module.

In Fig. 11b, PV-PEC system *J*–*V* curves are shown for three cases of BiVO_4_-coated WO_3_ placed in front of dual c-Si PV modules as in Fig. 11a. The *J*–*V* curves for the PV module are simulated in MATLAB using eqn (1) considering the light transmitted though the PEC photoanode and other optical losses. Transmittance spectra for all the PEC photoanodes were measured and plotted in Fig. S10.†*J*_sc_ is the calculated short-circuit current based on the solar spectrum and a conservative external quantum efficiency of 90%. *J*_0_ is the calculated exchange current, *R*_s_ is the series resistance, 0.001 kΩ cm^2^,^63^ and *R*_sh_ is the shunt resistance, 10 kΩ cm^2^,^64^ taken for a typical c-Si PV cell. *e* is the elemental charge, *n* is the ideality factor, *k* is Boltzmann's constant, and *T* is the temperature, taken as 297 K.1
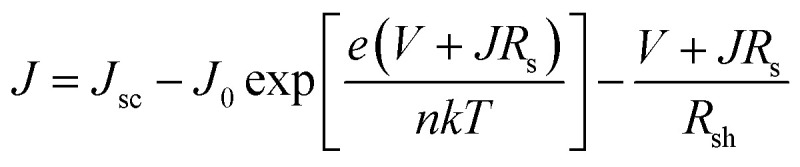


The operating points of the three tested PV-PEC systems in Fig. 11b are highlighted at the intersections between the three sets of PV and PEC *J*–*V* curves. The PV-PEC systems utilising a 2000 nm long WO_3_ NN photoanode coated with 100 nm thick BiVO_4_ and a 1000 nm long WO_3_ NN photoanode coated with 50 nm thick BiVO_4_ are simulated to have PV-PEC system operating currents of 0.5 mA cm^−2^ and 0.8 mA cm^−2^ respectively. The operating current for the simulated PV-PEC system with the 2000 nm long WO_3_ NN photoanode coated with 50 nm thick BiVO_4_ was an optimal value of 2.8 mA cm^−2^, which equates to an STH efficiency of 3.4%. This simulation shows the importance of increasing photocurrent from the PEC component and reflects the trends in system performance shown in Fig. 10 because the PV module in each case is providing an excess of photocurrent.

### 2.7 Comparison of similar unassisted PV-PEC systems in the literature

A wide range of unassisted PEC-based systems have been explored in the literature, including fully PEC^65,66^ and PV-PEC tandem devices. Herein, we focus exclusively on literature examples where WO_3_ and/or BiVO_4_ were applied as a photoanode in conjunction with a PV to achieve unassisted water splitting. Their performances are summarised in Table 2.

**Table 2:** Comparison of PV-PEC water splitting device performances from the literature focusing on metal oxide photoanode-based systems

PEC – PV	PEC synthesis	Biased J^a^ (mA cm^−2^)	SPP (mA cm^−2^)	Unassisted PV-PEC STH (%)	Ref.
WO_3_ – MAPI	AACVD	n.a.	1.24	0.75	34
WO_3_ – DSC	Sol–gel	2.4	2.23	3.1	67
BiVO_4_/CoPi – MAPI	MOD + PAE	2.2	n.a.	2.0	68
BiVO_4_/Ni:FeOOH – OPV	ED + PAE	1.9	n.a.	2.2	69
SiO_*x*_/BiVO_4_/Ni:FeOOH – MAPI	RIE + MOD + ED	6.0	n.a.	6.2	70
WO_3_/BiVO_4_/CoPi – GaAs/InGaAsP	GLAD + ED + ED	6.7	n.a.	8.1	19 and 68
TiO_2_/WO_3_/BiVO_4_/Fe(Ni)OOH – n-Si/SiO_2_	AACVD + spin coating	2.6	n.a.	0.31	27
W:BiVO_4_/Co-Pi – thin-film amorphous Si	Spray pyrolysis	4.0	n.a.	5.2	71
H, 1% Mo:BiVO_4_/NiFeO_*x*_ – crystalline silicon PV	Drop-casting & calcination	4.5	n.a.	3.0	72
WO_3_/BiVO_4_ – c-Si	AACVD + AACVD	n.a.	2.61	3.2	This work

a PEC only at 1.23 V *vs.* RHE and 1 sun illumination using solar simulated light; n.a. = not applicable.

Kafizas *et al.* produced nanoneedle-structured WO_3_ photoanodes using an analogous AACVD method to that applied in this work.^34^ Their highest performing material was composed of a 300 nm flat WO_3_ seed layer with a 5 μm top layer of WO_3_ nanoneedles. Under back irradiation, this material showed an SPP of 1.24 mA cm^−2^ at 1.23 V *vs.* RHE, and when coupled in tandem with a photovoltaic device containing a methylammonium lead iodide perovskite (MAPI), an STH of 0.75% for unassisted water splitting was predicted. Sivula *et al.* produced 2.5 μm thick mesoporous WO_3_ photoanodes through a sol–gel process that showed 2.4 mA cm^−2^ at 1.23 V *vs.* RHE and 1 sun irradiance.^67^ When coupled to a high open-circuit voltage cobalt-based dye-sensitized solar cell (DSC), it showed an unassisted STH of 3.1%.^67^

Kamat *et al.* demonstrated a tandem BiVO_4_ photoanode coupled with a MAPI PV that achieved STH efficiencies of 2.0%.^68^ Their BiVO_4_ was produced using metal organic deposition (MOD) and was coated with a cobalt phosphate (CoPi) surface co-catalyst using photo-assisted electrochemical (PAE) deposition.^68^ It resulted in a photocurrent density of 2.2 mA cm^−2^ at 1.23 V *vs.* RHE and 1 sun irradiance.^68^ Andrew *et al.* produced 700 nm thick mesoporous BiVO_4_ photoanodes using electrochemical deposition (ED) that were coated with a nickel : iron oxyhydroxide (Ni : FeOOH) co-catalyst using PAE deposition.^69^ Their photoanodes showed a photocurrent density of 1.9 mA cm^−2^ at 1.23 V *vs.* RHE and 1 sun irradiance, and when coupled with a non-fullerene acceptor organic photovoltaic (OPV) showed an unassisted STH of 2.2%.^69^ Using SiO_*x*_ nanocones, produced using a reactive ion etching process (RIE), Qiu *et al.* grew a conformal coating of Mo-doped BiVO_4_ on top using a MOD followed by a Ni : FeOOH surface co-catalyst using PAE deposition.^70^ Their photoanodes showed photocurrent density of 6.0 mA cm^−2^ at 1.23 V *vs.* RHE and 1 sun irradiance, and when coupled with a MAPI PV showed an unassisted STH of 6.2%.^70^ Lastly, the current state-of-the-art example, produced by Pihosh *et al.*, is a nanostructured WO_3_/BiVO_4_/CoPi system.^19^ 3 μm long WO_3_ nanorods were made using glancing angle deposition (GLAD) and were coated with BiVO_4_ and CoPi using ED methods.^19^ Their photoanodes showed a photocurrent density of 6.72 mA cm^−2^ at 1.23 V *vs.* RHE and 1 sun irradiance, and when coupled to a double-junction GaAs/InGaAsP PV showed an unassisted STH efficiency of 8.1%.^19^

Herein, we synthesised nanoneedle-structured WO_3_ with a conformal coating of BiVO_4_ using an AACVD method at ambient pressures (Fig. S1†), which simplifies the operation of and reduces the equipment and operating costs compared to many of the above-mentioned deposition techniques that require vacuum conditions. No surface co-catalyst as yet was applied in this work, which is often a pre-requisite for higher performance in this heterojunction system. Our best performing heterojunction was composed of 2000 nm long WO_3_ nanoneedles coated with a 50 nm thick conformal BiVO_4_ layer showing an SPP of 2.61 mA cm^−2^ under back irradiation, and when coupled to a dual c-Si PV in tandem, our modelling predicted an STH of 3.2%. Overall, the performance we observe is comparable to other examples from the literature. Notably, the higher performing examples use surface co-catalysts to achieve more favourable onset potentials and photocurrent densities in their photoanode, which was not explored herein but will be the subject of future work. Also, these examples that achieved higher performance used synthetic techniques that are not readily scalable (*e.g.* RIE, GLAD, *etc.*), and are therefore unlikely to be used in the commercial mass-production of this technology. Broadly, many of the PVs applied in these studies are either not commercially available (*e.g.* DSC, MAPI and OPV) or not cost effective (GaAs/InGaAsP). As such, the focus of this work was to use a scalable synthetic technique such as CVD, which is currently used to produce FTO-coated glass and can therefore be applied in line to produce photoanodes, alongside a PV material that is commercially available and low cost (*i.e.* c-Si). Fig. S1† shows a photograph of our CVD reactor which can deposit samples up to 16 cm × 5 cm surface area. A further publication currently in preparation will demonstrate the ability for our CVD method to produce arrays of photoanodes on a larger scale – increasing the size from the ∼1 cm^2^ to >50 cm^2^ scale – and couple these with c-Si PV in tandem to demonstrate unassisted water splitting at a scale more commensurate to its future commercial application.

## 3. Conclusions

BiVO_4_-coated WO_3_ nanostructured photoanodes for the water oxidation reaction were fabricated by aerosol-assisted chemical vapour deposition (AACVD) at atmospheric pressure, which is a considerable simplification and reduction in equipment costs compared to deposition under vacuum conditions. This work demonstrates the potential for AACVD to fabricate effective photoanodes for water splitting devices, despite not yet applying co-catalysts to our heterojunction system, which often results in higher performance. The mechanism of ternary BiVO_4_ deposition by CVD was investigated by thermogravimetric analysis of the metal–organic precursors used. TGA provided evidence of a difference in activation energy for decomposition between the precursors. XRD evidence of sub-stoichiometric phases of BiVO_4_ deposited at intermediate 300–350 °C temperatures allowed us to conclude that the stoichiometry of BiVO_4_ depends on the deposition temperature, rather than solely on the ratio of precursors in the starting deposition solution. CVD deposition of WO_3_/BiVO_4_ heterojunction photoanodes that showed photocurrents more than double compared to bare BiVO_4_ and several times that of bare WO_3_ photoanodes. The optimal solar predicted photocurrents of 1.9 mA cm^−2^ under front illumination and 2.6 mA cm^−2^ under back illumination for the samples tested represent promising STH conversion efficiencies of 2.3% and 3.2% respectively.

PV modules that can provide the 1.23 V *vs.* RHE to drive a PEC device include dual crystalline silicon cells connected electrically in series because they can absorb the red and near-infrared light, not utilised by the photoelectrode and are commercially available for a relatively low cost. We thus developed a model that simulates the performance of tandem PV-PEC devices, where our WO_3_/BiVO_4_ heterojunction photoanodes were coupled to a dual c-Si PV in tandem, and a maximum STH performance of 3.2% was predicted. Further increases in the PEC photocurrent and in the photovoltage provided by the PV module would shift the operating current to higher values. A further publication currently in preparation will demonstrate the ability for our CVD method to produce arrays of photoanodes on a larger scale (>50 cm^2^) more commensurate to commercial application. There exists a need to examine larger scale, unassisted water splitting devices, so that technoeconomic and life-cycle analyses can be more accurately conducted. Furthermore, additional steps may be implemented using AACVD, such as the incorporation of surface co-catalysts, to beneficially cathodically shift the onset potential and increase the plateau photocurrents to achieve higher overall STH efficiency.

## 4. Experimental section

### 4.1 Metal oxide fabrication by chemical vapour deposition

The metal–organic precursors tungsten hexacarbonyl (97%, Aldrich), bismuth triphenyl (98+%, Alfa Aesar), and vanadium(iii) acetylacetonate (97%, Aldrich) were used as received. FTO-coated glass substrates (1.33 × 2.5 cm^2^ × 0.22 cm thick) (TEC 15, Pilkington) were cleaned by successive sonication (VWR ultrasonic cleaner, 30 W, 45 kHz) in detergent with deionised water, deionised water, acetone, and isopropanol for 10 minutes each. The substrates were placed on a carbon block in a custom-built quartz tube reaction chamber and heated to the target deposition temperature. For WO_3_ deposition, varying volumes of 11.4 mM concentration precursor solution in 3 : 1 acetone and methanol was aerosolised by a humidifier (Liquifog®, Johnson Matthey, ∼1.6 MHz operating frequency) and carried into the reaction chamber by a 2 L min^−1^ flow of inert N_2_ gas. The carbon block was heated with an internal cartridge heater to 375 °C to grow nanoneedle films and to 325 °C to grow flat WO_3_ films as shown previously by Kafizas *et al.*^34^ Following complete transfer of precursor, the films were annealed in a 500 °C furnace (Nabertherm Electric Muffle Furnace, L 9/11/B410) in air for 2 hours (temperature ramp rate of 200 °C per hour), which leaves an optimal quantity of oxygen vacancies in the films.^37^ Deposition of BiVO_4_ occurred similarly with varying volumes of 5 mM concentration precursor solution aerosolised and carried into the reaction chamber by a 1 L min^−1^ flow of compressed air. The carbon block was heated to 400 °C during the deposition. Annealing of the BiVO_4_ was also done at 500 °C for 2 hours in air, which crystallises the BiVO_4_ with an optimal grain size while limiting vanadium vacancies that form trap states for recombination^73^ and reduces propensity to dissolve upon illumination in phosphate electrolyte solutions.^74^ Heterojunction samples were fabricated by sequentially depositing WO_3_, annealing at 500 °C for 2 hours, and depositing BiVO_4_ before a second annealing stage while adjusting the volumes of precursor solution transferred.

### 4.2 Physical characterisation

Scanning electron microscopy (SEM) images were taken with a Zeiss Auriga microscope with a 5 kV acceleration voltage, Inlens detector, and 5 mm working distance. Side-on SEM images of sample cross sections were used to determine that the WO_3_ nanoneedles deposited at a rate of 500 nm per 10 mL of precursor, BiVO_4_ deposited on nanoneedles at a rate of 25 nm per 10 mL of precursor, and 100 nm per 10 mL of BiVO_4_ precursor on flat surfaces. Thermo-gravimetry analysis with mass spectrometry (TGA-MS) was tested with a Mettler Toledo TGA/DSC 1LF/UMX, HIDEN ANALYTICAL HPR-20 QIC Evol. Precursor powders stored in air with initial masses of approximately 5 mg were tested under air gas environment at constant heating rate of 10 °C per minute. X-ray diffraction (XRD) patterns were measured with a Bruker D2 Phaser diffractometer with parallel beam optics equipped with a Lynx-Eye detector. X-rays were generated using a Cu source (*V* = 30 kV, *I* = 10 mA) through a 0.6 mm slit; with Cu K_α1_ (*λ* = 1.54056 Å) and Cu K_α2_ radiation (*λ* = 1.54439 Å) emitted with an intensity ratio of 2 : 1. Patterns were collected between 10° ≤ 2*θ* ≤ 70° with a step size of 0.0527° and a speed of 1 second per step. Patterns were compared to references from the Physical Sciences Data-Science (PSDS) database. Raman spectra were obtained using a Horiba LabRAM Infinity spectrometer equipped with a helium–neon laser (633 nm, 8 mW) from 100 to 1100 cm^−1^. X-ray photoelectron spectroscopy (XPS) between 0 and 1400 eV binding energy was measured with a Thermo Fisher K-Alpha+ automated system with Al Kα X-ray source and ion beam etch sputterer. Transmittance and reflectance of solid films were measured by a Shimadzu UV-2600 UV-vis spectrometer with integrating sphere in the range of 190 to 1200 nm with 1 nm resolution. Solution phase UV-vis spectroscopy was carried out on a Shimadzu 2600i spectrometer.

### 4.3 Photoelectrochemical experiments

A three-electrode set-up in a cappuccino-type cell^75,76^ was used in all experiments with a pH 7, 0.1 M aqueous potassium phosphate buffer electrolyte (potassium phosphate dibasic, ACS reagent ≥ 98%, Sigma-Aldrich & potassium phosphate monobasic, ACS reagent ≥ 99%, Sigma-Aldrich) made with 18.2 MΩ cm ultrapure water. Pt mesh and an Ag/AgCl (saturated KCl) electrode served as the counter and reference electrode respectively. Potentials *versus* the Ag/AgCl reference electrode were applied using a Metrohm Autolab PGSTAT 101 and converted to the reversible hydrogen electrode scale using the Nernst equation: *V*_RHE_ = *V*_Ag/AgCl_ + 0.0592 × pH + *V*^0^_Ag/AgCl_, for *V*^0^_Ag/AgCl_ = 0.197 V *vs.* RHE. Illumination was provided by a 75 W Xe lamp and monochromator (OBB-2001, Photon Technology International) and calibrated with a Thorlabs optical power meter with photodiode sensor (PM100D Power Energy Meter equipped with a S120VC sensor). The PEC performances are quantified by measuring the current–voltage (*J*–*V*) characteristic curves and the incident photon to current efficiency (IPCE) profiles. *J*–*V* Curves are obtained by linear sweep voltammetry at 10 mV s^−1^ under white light illumination by approximately 1 sun intensity as determined by measuring the photocurrent with a Si photodiode. IPCE values are obtained by changing the wavelength of calibrated monochromatic light while measuring the photocurrent by chronoamperometry under applied potential of 1.23 V *vs.* RHE. The IPCE was calculated using the following equation:

2 IPCE(%) = (*I*_ph_ × 1239.8)/(*P*_mono_ × *λ*) × 100

where *I*_ph_ is the photocurrent (mA cm^−2^), 1239.8 is the multiplication of Planck's constant with the speed of light (eV nm), *P*_mono_ is the light power at a given wavelength (mW cm^−2^) and *λ* is the wavelength of the monochromated light (nm). The solar water splitting activity was calculated using IPCE measurements. The solar predicted photocurrent (SPP) was determined by multiplying the IPCE with the AM1.5 solar spectrum and integrating over all wavelengths to determine the total current.3
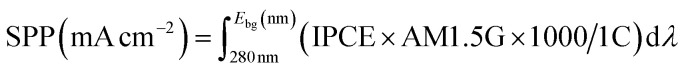
where AM1.5G is the solar photon flux (photons cm^−2^), *E*_bg_ is the material bandgap (517 nm) and 1C is 6.241 × 10^18^ electrons per second. The solar to hydrogen conversion efficiency (STH) was then calculated by the following equation:

4 STH(%) = SPP/81.3 × 100

where 81.3 in units of mA cm^−2^ is the theoretical photocurrent for complete conversion of the solar photon flux spectrum into a water splitting photocurrent.

Calculating AM1.5G photocurrents based on IPCE integration gives the same result as a photocurrent measured under solar simulated light when the measurements are conducted within a light intensity regime such that photocurrents change linearly with light intensity. For materials in which intensity-dependent recombination dominate, the photocurrent would increase non-linearly with light intensity. There is evidence in the literature that for moderate illumination intensities less than AM1.5G intensity, photocurrent densities do vary linearly with light intensity for BiVO_4_ and other metal oxides.^77,78^ This method additionally prevents adverse effects such as heating of the device and electrolyte. The measurement of IPCE profiles additionally gives the user insights into properties such as photoabsorber bandgap and losses for photons with short wavelengths due to illumination through the glass backing of photoelectrodes. IPCE integration is also important to highlight as an acceptable route to enabling the use of more inexpensive lighting (that otherwise would have a spectrum incongruent with the solar spectrum), to give results that then may be rigorously compared to AM1.5 photocurrents reported in the literature.

## Author contributions

Conceptualization, B. T., J. N., and A. K.; investigation, B. T. and S. P.; project administration, B. T.; supervision, J. N. and A. K.; visualization, B. T.; writing – original draft, B. T.; writing – review & editing, B. T., S. P., J. N., A. K.

## Conflicts of interest

The authors declare no conflicts of interest.

## Supplementary Material

SC-016-D4SC08595G-s001

## Data Availability

The data that support the findings of this study are available in the tables presented in the study and in the ESI.†
