# Supplementary material for: The scalable growth of high-performance nanostructured heterojunction photoanodes for applications in tandem photoelectrochemical-photovoltaic solar water splitting devices
Source: Chem Sci. 2025 Apr 1;16(18):7794–810. doi: 10.1039/d4sc08595g (PMC11976315; doi:10.1039/d4sc08595g)
Supplement: SC-016-D4SC08595G-s001 [file SC-016-D4SC08595G-s001.pdf]

Supplementary Information (SI) for Chemical Science.
This journal is © The Royal Society of Chemistry 2025

Supporting Information

The scalable growth of high-performance nanostructured heterojunction photoanodes for applications in tandem photoelectrochemical-photovoltaic solar water splitting devices

Brian Tam,[a,b*] Sebastian Pike,[c] Jenny Nelson[b] and Andreas Kafizas[a,d]

[a]*Department of Chemistry, Molecular Science Research Hub, Imperial College London, White City, London, W12 0BZ, U.K.*

[b]*Department of Physics, Imperial College London, South Kensington, London, SW7 2AZ, U.K.*

[c]*Department of Chemistry, University of Warwick, Coventry, CV4 7AL, U.K.*

[d]*London Centre for Nanotechnology, Imperial College London, South Kensington, London, SW7 2AZ, U.K.*

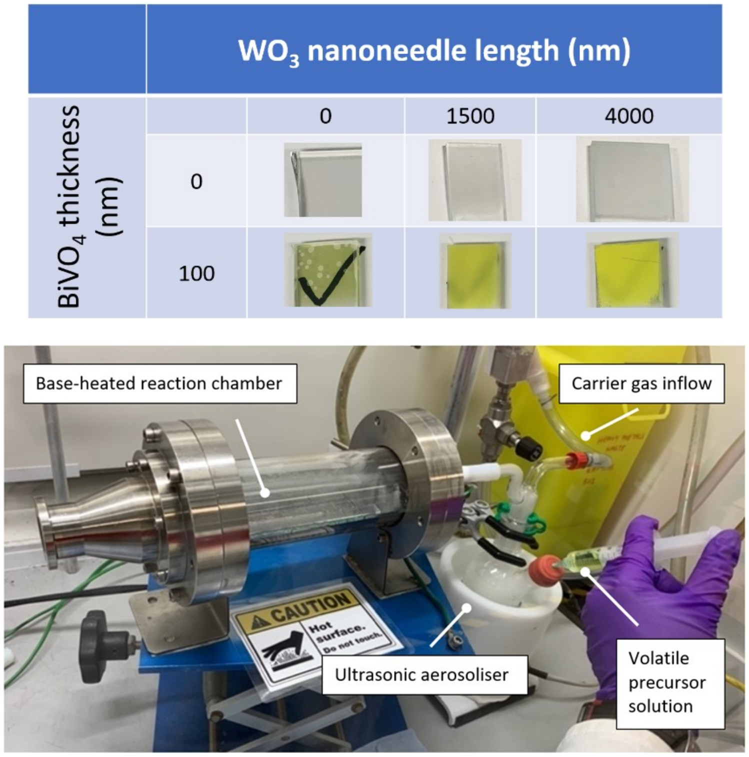

Figure S1: Photographs of representative WO_3, $BiVO_4$ and heterojunction thin films on FTO-coated glass. Samples with $BiVO_4$ are photographed with a black check-mark underneath for contrast. Photograph of atmospheric-pressure CVD reactor with a total surface area of 5 cm x 16 cm.

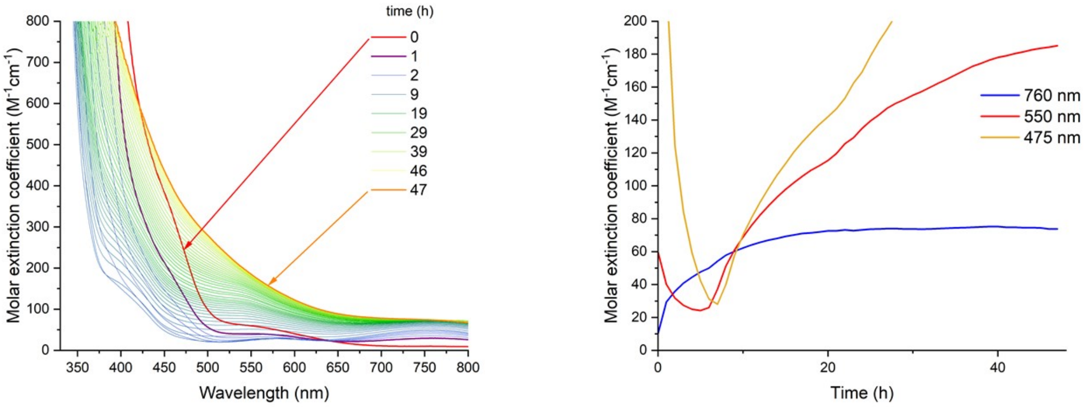

Figure S2: a) UV-vis spectra of a 5 mM solution of $V(acac)_3$ + $BiPh_3$ in 3:1 acetone:methanol over time. The $V(acac)_3$ was stored in a N_2 filled glovebox, and the first spectrum was collected at '0' hours immediately following exposure to air and rapid solvation in the prepared solution. The solution changes colour from yellow to green over several hours before changing further to orange after two days. b) Plot showing the absorption at specific wavelengths over time. The

peak at 760 nm is indicative of the growth of V(IV) species (e.g. $VO(acac)_2$). This reaches the approximate extinction coefficient expected for ~5 mM $VO(acac)_2$, suggesting a significant oxidation to V(IV) occurs within one day. Furthermore, the intensity at 475 nm and 550 nm display the consumption of V(III) over the first six hours which is then outweighed by growth of V(V) species which also absorb in this region (N.B. $VO(acac)_2$ has negligible extinction coefficient at 475 nm). Under these conditions, significant V(IV) is produced within a few hours of exposure to air, further oxidation to V(V) compounds occurs after ~5h exposure to air.

Note that the solution environment is important for the kinetics of this process, with MeOH important for rapid oxidation. $V(acac)_3$ dissolved in $CHCl_3$ (orange solution) reacts slowly under air (days) to generate $VO(acac)_2$ & acac-H (green solution), confirmed by X-ray crystallography of the blue/green crystals grown from this solution. Similar colour changes are observed in acetone solution. Addition of MeOH to green partially oxidised solutions results in a rapid colour change to brown/orange indicating methanol enables the second oxidation to V(V) species.

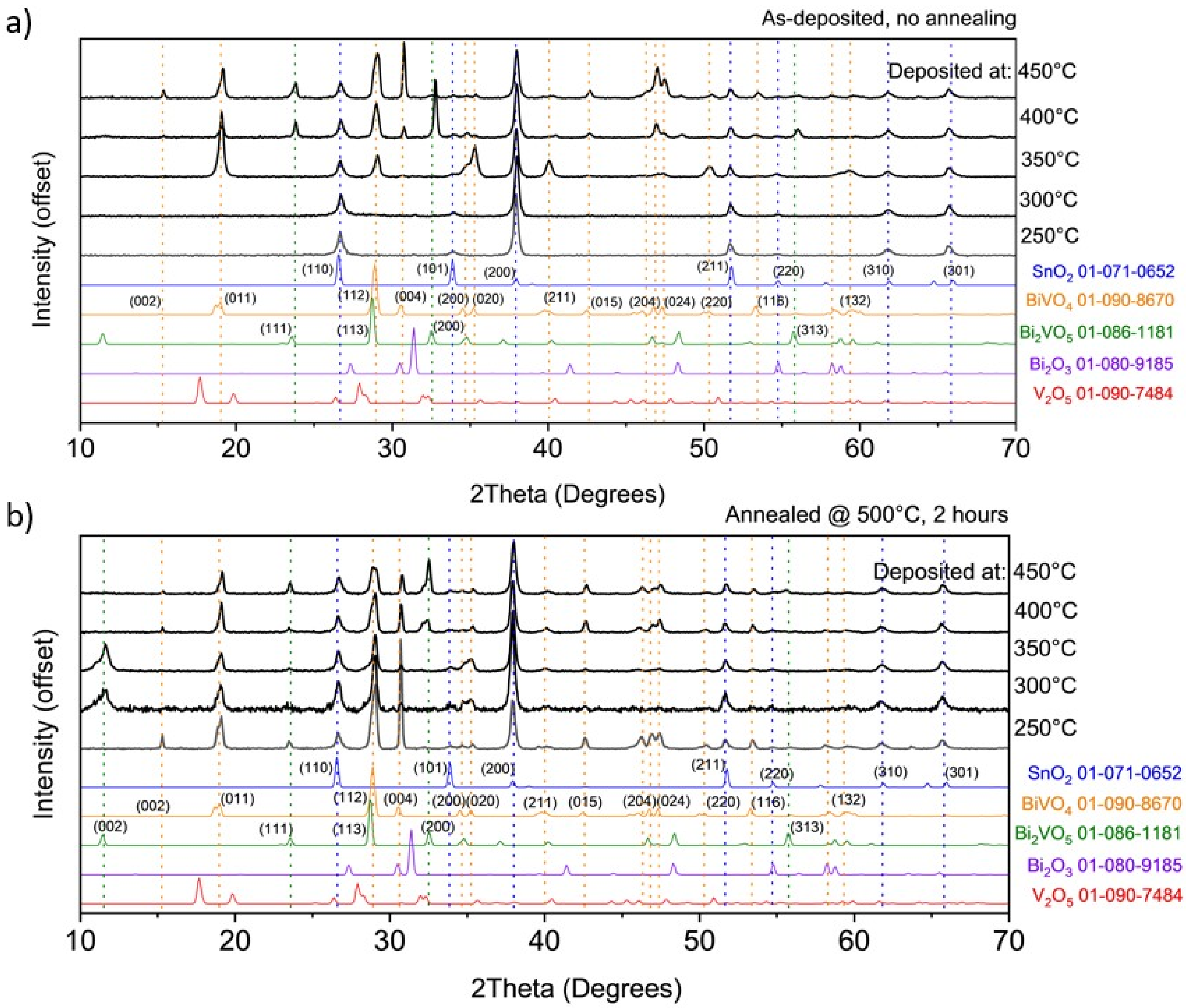

Figure S3: XRD patterns for $BiVO_4$ films grown on FTO-coated glass by AACVD at a range of temperatures at which a) the films are as deposited, and b) the films are subsequently annealed at 500°C for 2 hours in air. The vanadium precursor was aged for 1 month and subsequently a minute amount of Bi_2VO_5 is present in all samples measured.

In Figure S3a, the samples deposited at lower temperatures of 250°C and 300°C only show the (110) (101) (200) (211) (220) (310) & (301) diffraction peaks for the FTO substrate (PDF no. 01-071-0652), in agreement with the low levels of decomposition observed in the TGA-MS profiles of the $Bi(Ph)_3$ and $V(acac)_3$ precursors. Samples deposited at higher temperatures of 350°C, 400°C and 450°C show the (002) (011) (112) (004) (200) (020) (211) (015) (204) (024) (220) (116) & (132) peaks largely corresponding to polycrystalline $BiVO_4$ (PDF no. 01-090-8670) and a minority of peaks (111) (200) & (313) of Bi_2VO_5 (PDF no. 01-086-1181). In Figure S3b, the XRD patterns of all samples have the full breadth of peaks corresponding to $BiVO_4$, although the samples deposited at 300°C and 350°C also have large peaks corresponding to Bi_2VO_5 (002) plane. At higher temperatures there are also increasing proportions of the Bi_2VO_5 (200) plane.

Table S1: XPS work function measurements

	Work function before etching (eV)^	C1s peak height before etching (counts)*	Work function after twice etching (eV)^	C1s peak height after twice etching (counts)*
WO_3 spot 1	4.3 (4.28)	5500	5.3 (5.26)	Noise, <500
WO_3 spot 2	4.5 (4.45)	5500	4.5 (4.50)	3000
WO_3 spot 3	4.3 (4.34)	5500	4.7 (4.67)	2000
WO_3 spot 4	4.3 (4.34)	5500	4.7 (4.66)	2000
**Average WO_3**	**4.3**		**4.8**	
$BiVO_4$ spot 1	4.4 (4.42)	4500	4.9 (4.86)	Noise, <500
$BiVO_4$ spot 2	4.6 (4.55)	4500	4.7 (4.72)	1000
**Average $BiVO_4$**	**4.5**		**4.8**	
$BiVO_4$-WO_3 spot 1	4.5 (4.53)	3000	4.6 (4.63)	1000
$BiVO_4$-WO_3 spot 2	4.5 (4.45)	3500	4.6 (4.59)	1500
**Average $BiVO_4$-WO_3**	**4.5**		**4.6**	

*C1s scans – Mode: CAE, Pass Energy: 20 eV, Number of scans: 2, Dwell Time: 50 ms, Energy Step Size: 0.1 eV, 191 Energy Channels

^Work function Cut off scans – Mode: CAE, Pass Energy: 10 eV, Number of Scans: 1, Dwell Time: 75 ms, Energy Step Size: 0.050 eV, 121 Energy Channels

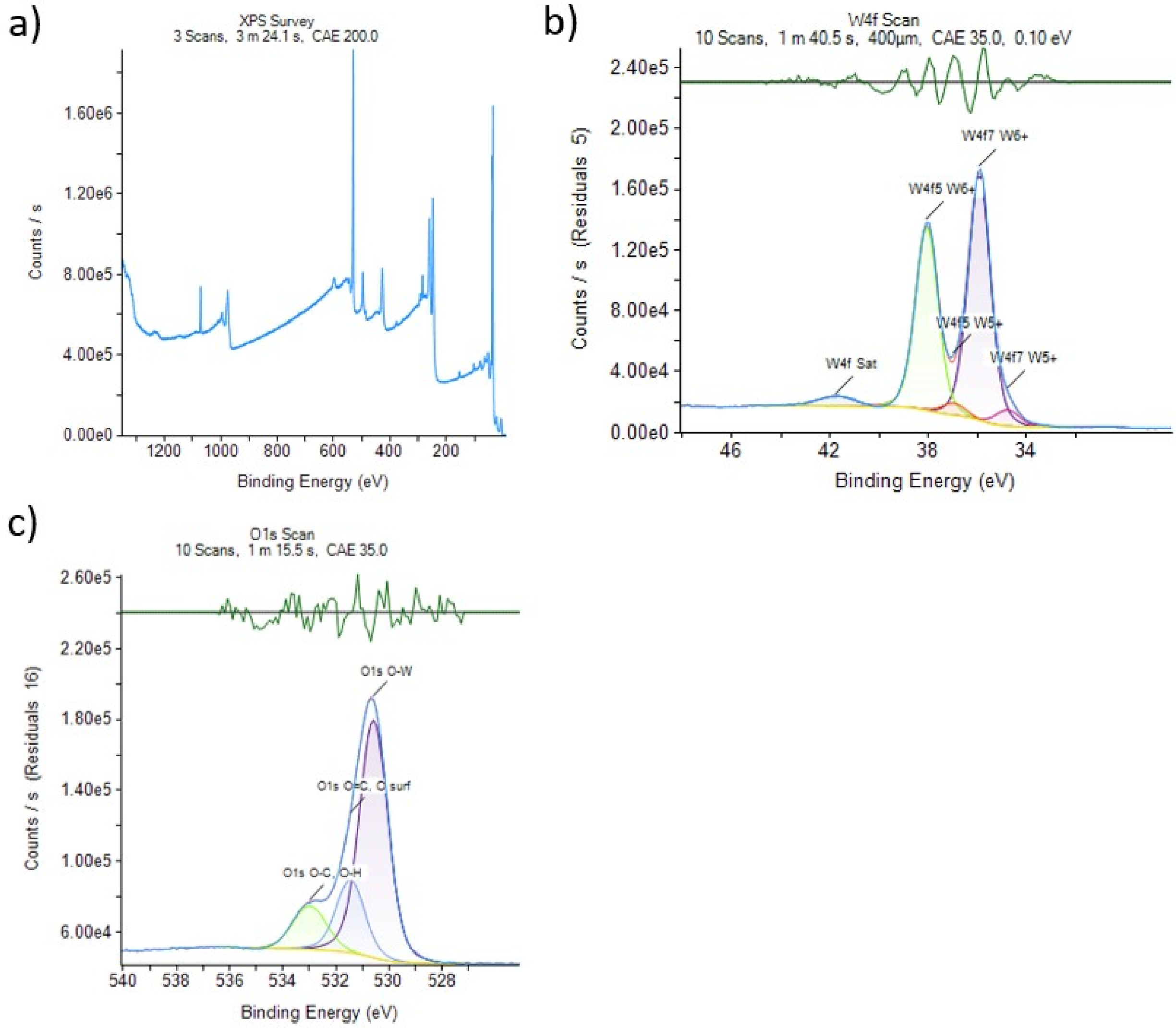

Elemental ID and Quantification

Name	*Peak BE*	*FWHM eV*	*Area (P) CPS.eV*	*Atomic %*
W $4f_{7/2}$ W^{6+}	35.92	1.09	194321.37	25.43
W $4f_{7/2}$ W^{5+}	34.77	1.14	13386.16	1.75
O 1s O-W	530.58	1.26	188333.22	72.82

Figure S4: XPS peak fitting of WO_3 on FTO, a) survey scan, b) W peaks, c) O peaks, and elemental quantification

Table S2: XPS peak fit parameters for WO_3 on FTO

Ref.	Name	Peak BE	Height CPS	Height Ratio	Area CPS.eV	Area Ratio	FWHM fit param (eV)	L/G Mix (%) Convolve	Tail Mix (%)	Tail Height (%)	Tail Exponent
A	W $4f_{7/2}$ WO_3	35.92	160473.2 5	1	193985.45	1	1.09	19.5	100	0	0
							0.5 : 3.5	fixed	fixed	fixed	fixed
B	W $4f_{5/2}$ WO_3	38.06	120354.9 4	0.75	145489.09	0.75	1.09	19.5	100	0	0
		A+2.17 (+0.2 -0.1)	A*0.75				A*1	A*1	A*1	A*1	A*1
C	W4f Sat	41.76	6864.39	0.04	12929.04	0.07	1.69	20	100	0	0
							0.5 : 3.5	fixed	fixed	fixed	fixed
D	W $4f_{7/2}$ WO_2	34.8	10740.56	0.07	13587.81	0.07	1.14	19.5	100	0	0
							A*1 (±0.05)	A*1	fixed	fixed	fixed
E	W $4f_{5/2}$ WO_2	36.87	8055.42	0.05	10190.85	0.05	1.14	19.5	100	0	0
		D+2.17 (+0.2 -0.1)	D*0.75				D*1	D*1	D*1	D*1	D*1
Ref.	Name	Peak BE	Height CPS	Height Ratio	Area CPS.eV	Area Ratio	FWHM fit param (eV)	L/G Mix (%) Convolve	Tail Mix (%)	Tail Height (%)	Tail Exponent
N	O1s O-W	530.58	133777.3 5	1	188333.22	1	1.26	20	100	0	0
							0.5 : 3.5	fixed	fixed	fixed	fixed
O	O1s O-C, O-H	532.98	24392.61	0.18	39117.12	0.21	1.51	10	100	0	0
							0.5 : 3.5	0.461806	fixed	fixed	fixed
P	O1s O=C,	531.45	41021.53	0.31	57750.56	0.31	1.26	20	100	0	0

	O surf										
							N*1	N*1	fixed	fixed	fixed
Ref.	Name	Peak BE	Height CPS	Height Ratio	Area CPS.eV	Area Ratio	FWHM fit param (eV)	L/G Mix (%) Convolve	Tail Mix (%)	Tail Height (%)	Tail Exponent
F	C1s C-C	285.12	7021.83	1	11513.82	1	1.47	20	100	0	0
							0.5 : 3.5	fixed	fixed	fixed	fixed
G	C1s C-O	286.19	982.19	0.14	1610.52	0.14	1.47	20	100	0	0
		285.99 : 286.19					F*1	F*1	fixed	fixed	fixed
H	C1s C=O	287.15	609.46	0.09	999.35	0.09	1.47	20	100	0	0
							F*1	F*1	fixed	fixed	fixed
I	C1s O-C-O	288.82	1001.63	0.14	1642.4	0.14	1.47	20	100	0	0
							F*1	F*1	fixed	fixed	fixed

Binding energy in table referenced to adventitious C1s, C-C peak at 285.12 eV, subtract by 0.32 eV to obtain binding energy referenced to 284.8 eV.

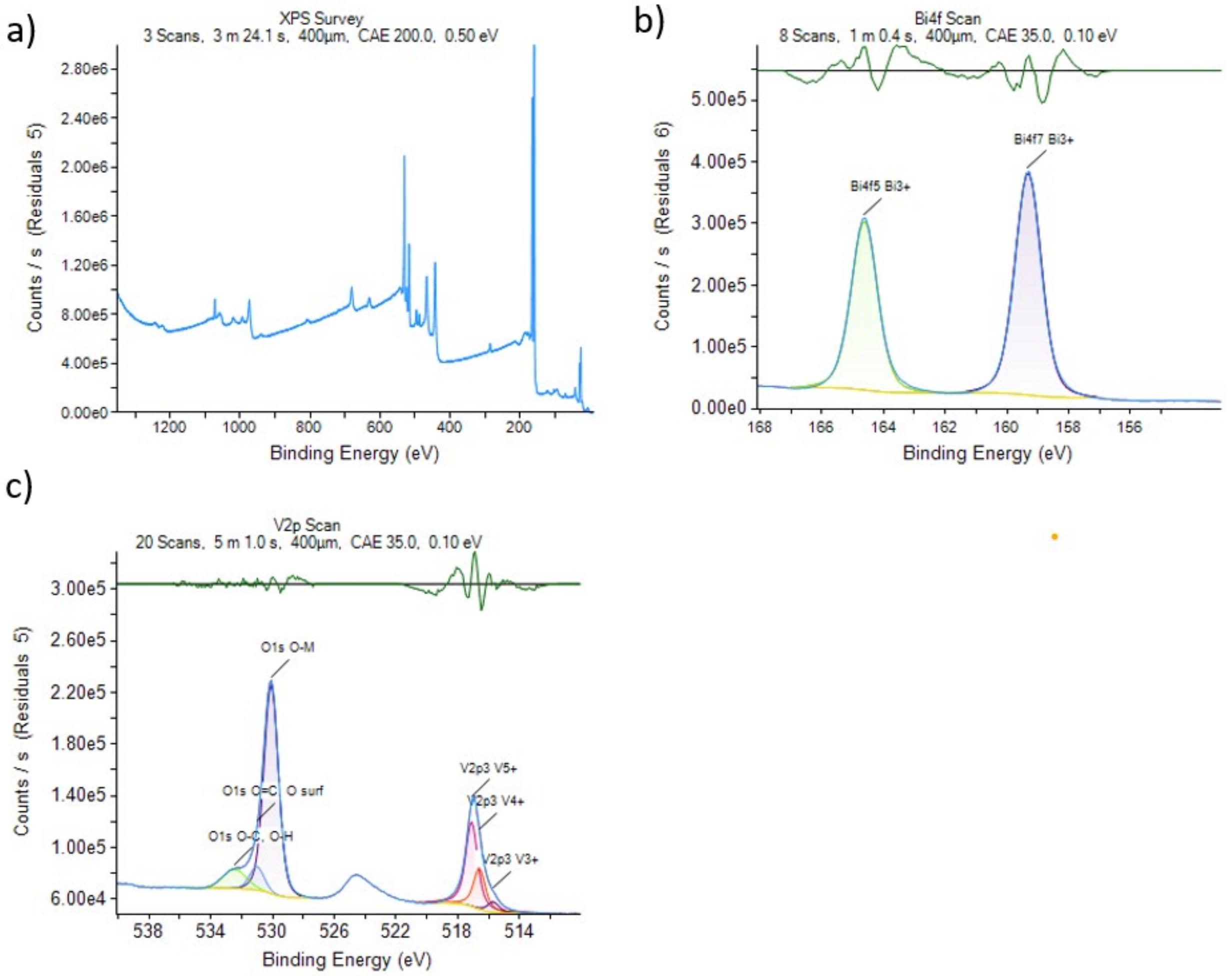

Elemental ID and Quantification

Name	*Peak BE*	*FWHM eV*	*Area (P) CPS.eV*	*Atomic %*	*Q*
Bi4f7 Bi3+	159.31	1.01	408915.94	14.24	1
O1s O-M	530.09	1.11	199867.86	66.50	1
V2p3 V5+	517.10	0.92	77515.87	12.02	1
V2p3 V4+	516.60	0.92	37275.41	5.78	1
V2p3 V3+	515.70	0.92	9410.36	1.46	1

Figure S5: XPS peak fitting of $BiVO_4$ on FTO, a) survey scan, b) Bi peaks, c) O & V peaks, and elemental quantification

Table S3: XPS peak fit parameters for $BiVO_4$ on FTO

Ref.	Name	Peak BE	Height CPS	Height Ratio	Area CPS.eV	Area Ratio	FWHM fit param (eV)	L/G Mix (%) Convolve	Tail Mix (%)	Tail Height (%)	Tail Exponent
A	Bi $4f_{7/2}$ Scan A	159.31	360753.1	1	408915.9	1	1.01	22.13	100	0	0
							0.5 : 3.5	0.461806	fixed	fixed	fixed
B	Bi $4f_{5/2}$ Scan A	164.62	274172.3	0.76	310776.1	0.76	1.01	22.13	100	0	0
		A+5.30 (+0.3 - 0.2)	A*0.76				A*1	A*1	A*1	A*1	A*1
Ref.	Name	Peak BE	Height CPS	Height Ratio	Area CPS.eV	Area Ratio	FWHM fit param (eV)	L/G Mix (%) Convolve	Tail Mix (%)	Tail Height (%)	Tail Exponent
P	O 1s O-W	530.09	161436.9	1	199867.9	1	1.11	20	100	0	0
							0.5 : 3.5	fixed	fixed	fixed	fixed
Q	O 1s O-C, O-H	532.45	14827.16	0.09	28127.25	0.14	1.78	10	100	0	0
							0.5 : 3.5	0.461806	fixed	fixed	fixed
R	O 1s O=C, O surf	531.01	18117.12	0.11	22430	0.11	1.11	20	100	0	0
							P*1	P*1	fixed	fixed	fixed
S	V $2p_{3/2}$ Scan A	517.1	65675.36	0.41	77515.87	0.39	0.91	48.44	100	0	0
							0.5 : 3.5	0.461806	fixed	fixed	fixed
T	V $2p_{3/2}$	516.6	31581.61	0.2	37275.41	0.19	0.91	48.44	100	0	0

	Scan B										
		S-0.70 (±0.2)					S*1	S*1	fixed	fixed	fixed
U	V 2p Scan C	515.7	7972.94	0.05	9410.36	0.05	0.91	48.44	100	0	0
		T-0.70 (±0.2)					S*1	S*1	fixed	fixed	fixed

Binding energy in table referenced to adventitious C1s, C-C peak at 285.12 eV, subtract by 0.32 eV to obtain binding energy referenced to 284.8 eV.

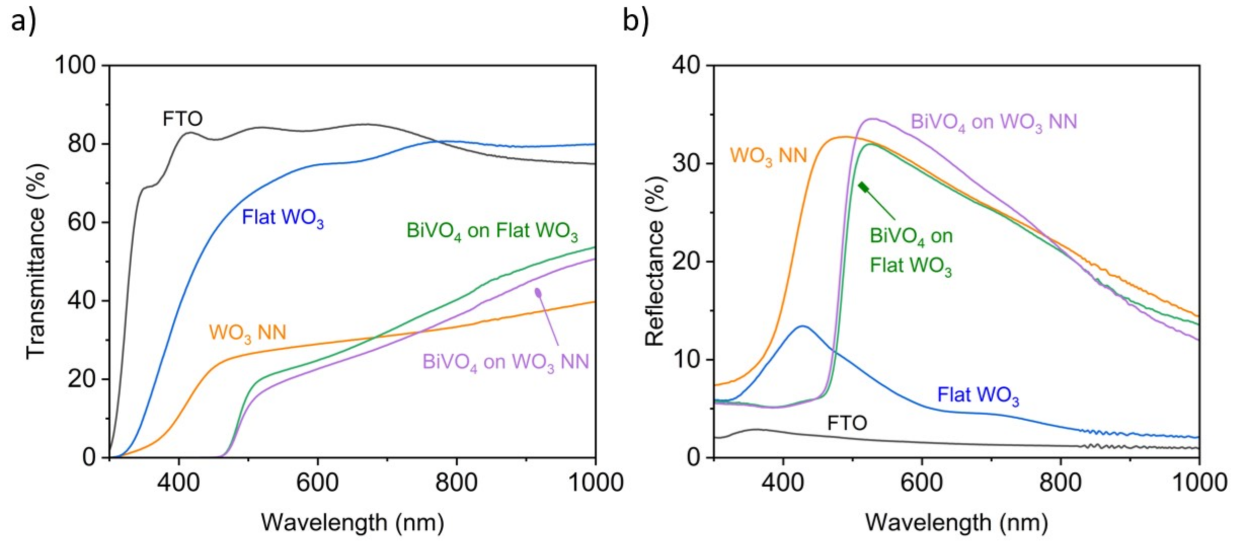

Figure S6: UV-visible spectroscopy comparing, a) transmittance, and b) total reflectance of representative samples.

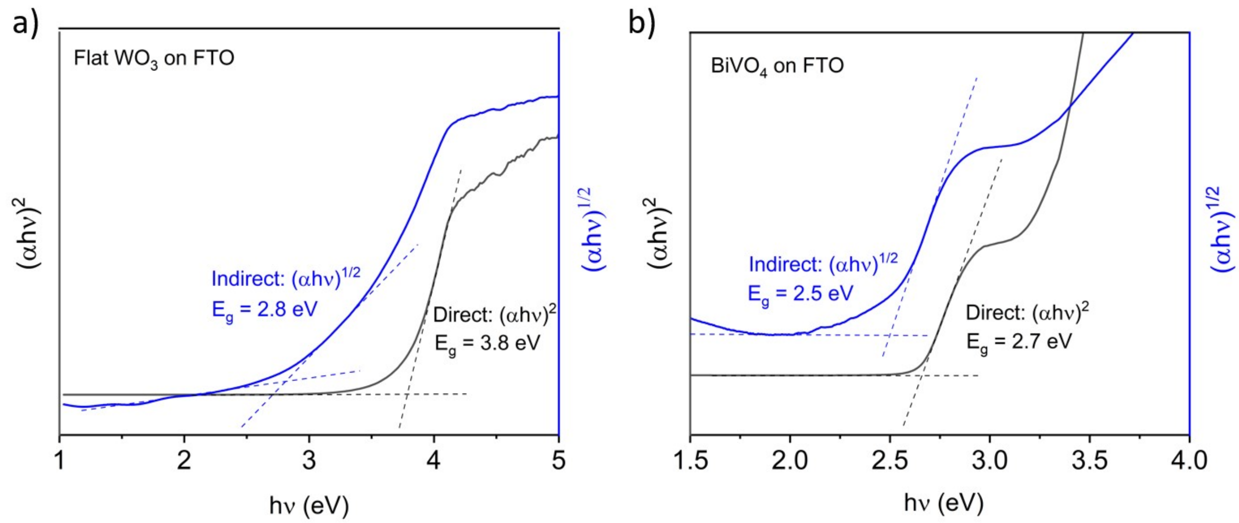

Figure S7: Tauc plots derived from UV-visible spectroscopy data comparing, a) flat WO_3 on FTO, and b) $BiVO_4$ on FTO

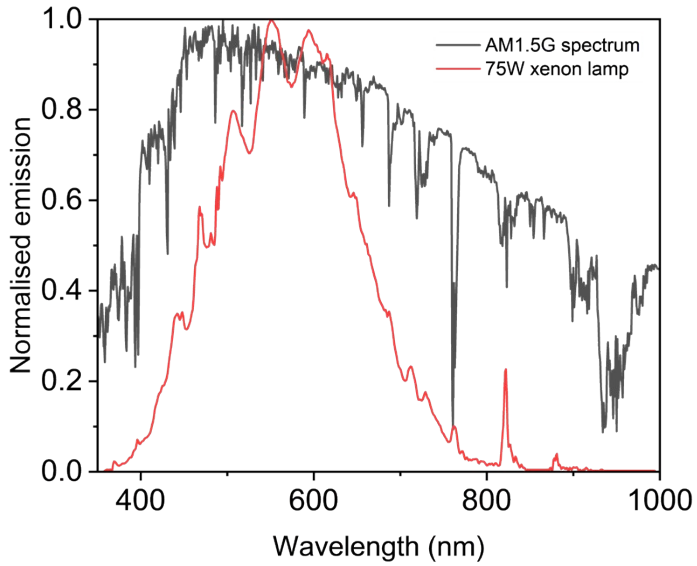

Figure S8. Normalised emission for the AM1.5G solar spectrum from NREL and the spectrum of the 75 W xenon lamp used in the laboratory with a KG3 (short-pass, heat protection) filter measured with an Ocean Optics spectrometer.

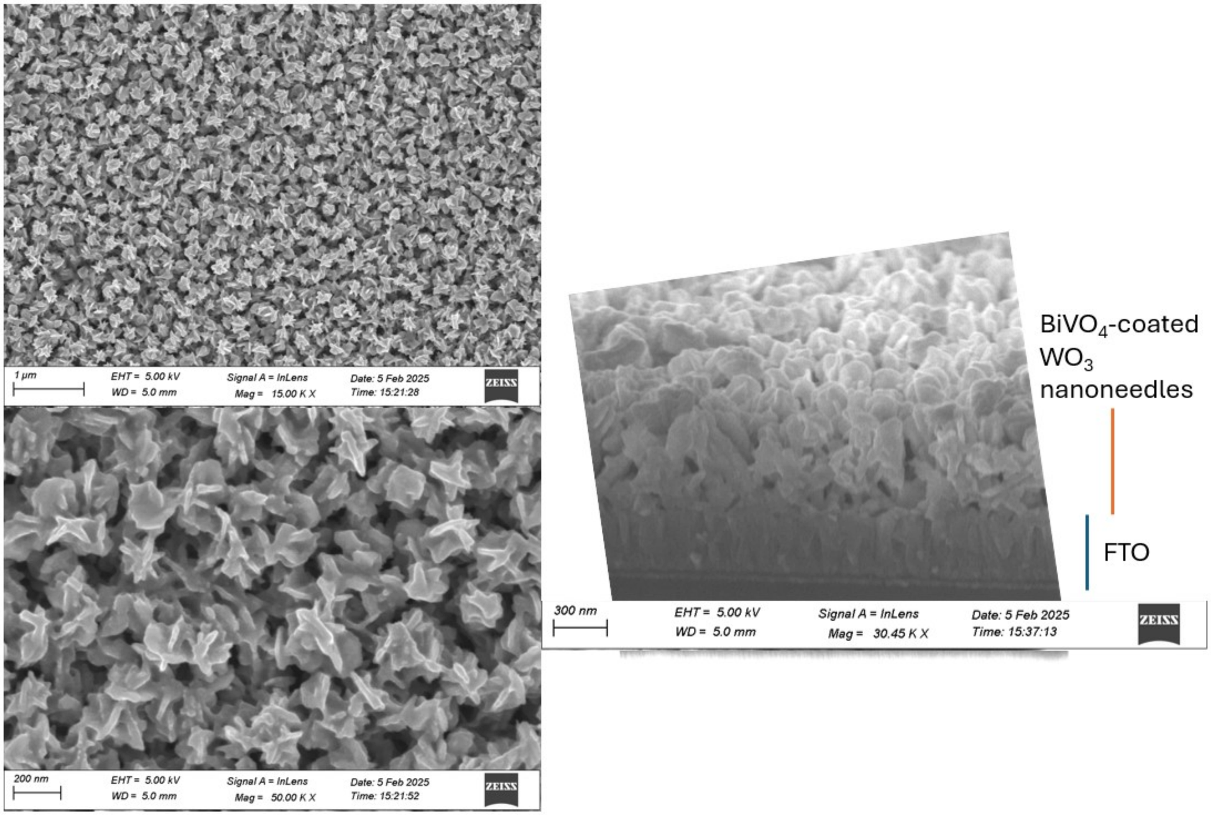

Figure S9: Top-down and side-on SEM images of $BiVO_4$-coated nanoneedles (roughly 500-600 nm in thickness) on FTO-coated glass (400 nm in thickness) after long-term photo(electro)chemical testing

Modelling a PV-PEC system: a dual c-Si PV solar cell placed behind a $BiVO_4$-coated WO_3 photoanode-based PEC module.

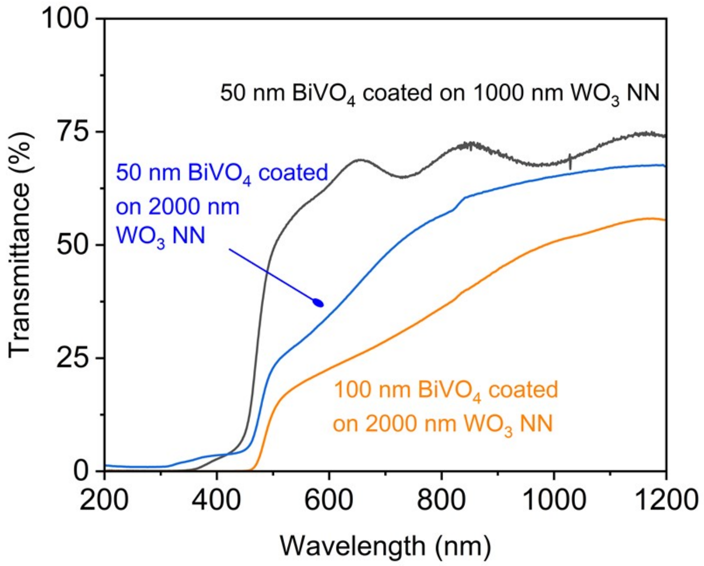

Figure S10: Measured transmittance spectra of PEC photoanodes used in **Figure 11b**

Code for simulating the dual crystalline-Si PV solar cell

The MATLAB code and supporting functions for simulating the PV J-V curves are readily available on Github at https://github.com/bsmtam/PV-JV.

Table of Contents figure:

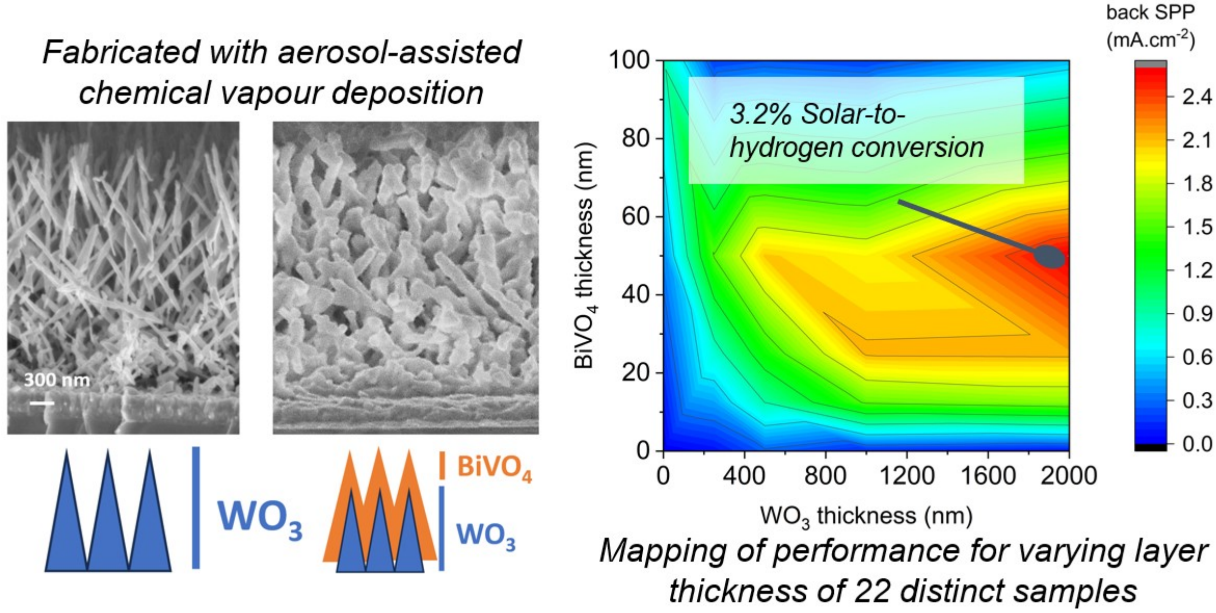

WO_3/ $BiVO_4$ heterojunction layer space systematically explored using chemical vapour deposition synthesis to map and identify samples of highest performance for solar water splitting applications.
